# Montmorillonite nanoclay as a sustainable adsorbent for removal of Acid Blue 113 dye from textile industrial effluent

**DOI:** 10.1038/s41598-026-50450-0

**Published:** 2026-05-11

**Authors:** Mohammed A. H. Dhaif Allah, Syed Noeman Taqui, Camellia Doroody, Kiran Shahapurkar, Syida Aameera Yakuth, Mohammad Nur-E-Alam, M. A. Umarfarooq, Andrey Pisarev, Razia Sulthana, Rayees Afzal Mir, Mahaboob Patel, S. Ramesh, Akheel Ahmed Syed

**Affiliations:** 1 Department of Agriculture, Faculty of Agriculture and Veterinary Medicine, Thamar University, Dhamar, Republic of Yemen; 2 Department of Studies in Chemistry, Bharathi College - Post Graduate and Research Centre, Bharathi Nagara, Karnataka, 571422 India; 3 Centre for Promotion of Research, Graphic Era (Deemed to Be University), Clement Town, Dehradun, India; 4 Centre for Research Impact and Outcome, Chitkara University Institute of Engineering and Technology, Chitkara University, Rajpura, Punjab 140401 India; 5 Center for Innovation and Inclusive Research, Sharda University, Greater Noida, Uttar Pradesh India; 6 Center of Excellence - Advanced Material Synthesis, Department of Mechanical Engineering, Alliance School of Applied Engineering, Alliance University, Bengaluru, 562106 India; 7 Department of Studies in Chemistry, University of Mysore, Manasa Gangothri, Mysore, Karnataka 570006 India; 8 Space Science Centre, Institute of Climate Change, Universiti Kebangsaan Malaysia, 43600 Bangi, Selangor Malaysia; 9 Department of Mechanical Engineering, Center for Material Science, Karpagam Academy of Higher Education, Coimbatore, Tamil Nadu India; 10 Faculty of Engineering, Lishui University, Lishui, 323000 Zhejiang China; 11 Maya School of Agriculture and Technology, Maya Devi University, Dehradun, Uttarakhand 248011 India; 12 Department of Mechanical Engineering, Wolaita Sodo University, 4620 Wolaita Sodo, Ethiopia; 13 Center of Advanced Manufacturing and Materials Processing (AMMP), Faculty of Engineering, University of Malaya, Kuala Lumpur, Malaysia; 14 Centre for Advanced Research and Innovation, Glocal University, Delhi-Yamunotri Marg, SH - 57, Mirzapur Pole, Saharanpur, Uttar Pradesh 247121 India

**Keywords:** Montmorillonite nanoclay, Acid Blue 113, Circular economy, Water footprint, Textile industrial effluent, Chemistry, Engineering, Environmental sciences, Materials science

## Abstract

This study investigates the application of Montmorillonite Nanoclay (MNC) as a sustainable, low-cost, and scalable adsorbent for removing the bisazo dye Acid Blue 113 (AB113) from aqueous solutions and textile industrial effluent (TIE). Considering the mutagenic potential of azo dye degradation products, the development of efficient and environmentally compatible remediation strategies is essential. A notable finding is the minimal influence of pH (2–12) and temperature (30–50 °C) on adsorption performance, demonstrating the robustness and practical applicability of the process. The effects of key operational parameters—including initial dye concentration (25–200 mg L^−1^), contact time (15–180 min), and adsorbent dosage (0.5–6.0 g L^−1^), were systematically evaluated. Adsorption equilibrium was analysed using various isotherm models, with the Vieth–Sladek model showing the best fit, while kinetic analysis indicated that the pseudo-second-order model governed the adsorption process. Diffusion modelling (film diffusion, Weber–Morris, and Dumwald–Wagner) revealed that mass transfer mechanisms predominated over intraparticle diffusion. Thermodynamic evaluation based on ΔG°, ΔH°, and ΔS° confirmed the feasibility and nature of adsorption. A two-level fractional factorial experimental design (FFED) combined with multiple regression analysis identified significant variables and optimised the process. Under optimal conditions (pH 7, adsorbent dosage 0.5 g L^−1^, initial dye concentration 858 mg L^−1^, contact time 204 min, 30 °C), a maximum adsorption capacity of 589 mg g^−1^ was achieved. These results demonstrate that MNC is an efficient and resilient adsorbent for AB113 removal and supports circular economy strategies through valorisation of dye-loaded sludge into composite materials.

## Introduction

The rapid expansion of the global textile industry, valued at USD 1,837.27 billion in 2023 and projected to grow at a compound annual growth rate (CAGR) of 7.4% between 2024 and 2030, has intensified concerns regarding its environmental footprint^1^. Among the most pressing challenges is the large-scale discharge of dye-laden effluents into aquatic systems, posing severe ecological and public health risks. While sustainable clothing initiatives are gaining momentum, the continued reliance on synthetic dyes presents a paradox that undermines environmental sustainability.

Synthetic dyes, particularly azo dyes, dominate the textile sector due to their low cost, ease of synthesis, and wide chromatic range^2,3^. Azo dyes account for approximately 60–70% of the total dye market, making them the most widely used class of colorants. However, their environmental persistence and toxicological implications are significant. The azo bond (–N=N–), a key structural feature, can undergo reductive cleavage by azo reductase enzymes, yielding aromatic amines. Many of these degradation products are toxic, mutagenic, and potentially carcinogenic^4^. Notably, certain aromatic amines derived from azo dyes remain insufficiently regulated, raising serious concerns about long-term exposure risks.

Acid Blue 113 (AB113), a representative bisazo dye, is extensively used in textile processing and is frequently detected in industrial effluents. Its complex structure, high stability, and resistance to biodegradation make it particularly challenging to remove using conventional treatment methods.

A wide range of remediation strategies has been explored for removing AB113 from aqueous systems. These include biological and hybrid bio-chemical processes^5–9^, electrocoagulation^10^, advanced oxidation processes such as UV-based degradation^11–15^, photocatalysis^16,17^, ultrasound-assisted degradation^18^, nanomaterial-based treatments^19^, and adsorption using inorganic materials such as activated carbon^20–25^. While these methods demonstrate varying degrees of effectiveness, they suffer from several limitations, including high operational costs, generation of secondary pollutants, sludge disposal issues, sensitivity to wastewater composition, and challenges in regeneration and scalability^26,27^.

In this context, adsorption has emerged as a promising and versatile technique due to its operational simplicity, high efficiency, and adaptability from laboratory to industrial scale. The effectiveness of adsorption largely depends on the nature of the adsorbent, necessitating the development of low-cost, sustainable, and high-performance materials. An ideal adsorbent should be abundantly available, economically viable, structurally porous, and capable of efficient contaminant removal without requiring extensive pre-treatment.

MNC is an important component of bentonite, has attracted significant attention owing to its layered structure, high surface area, and ion-exchange capacity. It has been widely investigated for applications in environmental remediation, pharmaceuticals, catalysis, and biomedical engineering^28–33^. In particular, its potential as an adsorbent for both organic dyes and heavy metals highlights its versatility and environmental compatibility^34–38^.

The performance of MNC for the removal of Acid Blue 113 (AB113) was compared with previously reported adsorbents to evaluate its relative efficiency and practical applicability.

The comparative analysis indicates that MNC exhibits competitive adsorption performance relative to many conventional and advanced adsorbents reported for AB113 removal. While some materials demonstrate comparable or higher adsorption capacities under optimised conditions, they often require strict pH control, chemical modification, or high operational costs. In contrast, MNC demonstrates a unique advantage in its operational robustness, maintaining consistent adsorption performance across a wide pH (2–12) and temperature (30–50 °C) range.

Furthermore, unlike engineered nanomaterials or chemically modified adsorbents, MNC is naturally abundant, cost-effective, and requires minimal pre-treatment, making it highly suitable for large-scale applications. An additional distinguishing feature of the present study is the integration of circular economy principles, wherein dye-loaded MNC is valorised into composite materials, addressing the critical issue of secondary waste management.

Overall, this comparison highlights that the strength of the present system lies not only in adsorption capacity but also in its simplicity, sustainability, and scalability, which are essential for real-world wastewater treatment applications.

Despite these advantages, limited studies have systematically explored the application of MNC for the removal of Acid Blue 113 from real textile industrial effluents, particularly with an integrated focus on post-treatment waste management. Furthermore, the fate of dye-laden adsorbents (sludge) and their potential valorisation remain underexplored in the context of sustainable remediation.

Therefore, the present study aims to investigate the efficacy of MNC as a sustainable adsorbent for the removal of AB113 from aqueous solutions and textile industrial effluents. In addition, this work explores an innovative approach for the reutilization of dye-adsorbed MNC sludge as reinforcing filler in polymer composite materials derived from plastic waste. This integrated strategy not only enhances dye remediation efficiency but also aligns with the principles of the circular economy by converting waste into value-added materials^39^.

## Experimental

### Materials

All chemicals and materials used in this study were procured from reliable local suppliers to evaluate their applicability under practical conditions. The use of commercially available reagents was intended to simulate realistic operational scenarios and ensure the feasibility of large-scale applications. Acid Blue 113 (AB113) dye was used as the target pollutant for adsorption experiments, while montmorillonite nanoclay (MNC) served as the adsorbent material. All chemicals were used as received without any further purification. Deionized water was used throughout the experiments for the preparation of dye solutions and washing procedures to maintain experimental consistency.

### Adsorbent research and description

#### Analysis of commercial sample of Acid Blue 113 purity

The commercial azo dyes often contain synthetic chemicals, byproducts, incomplete reaction intermediates, and starting materials. The existing components in the mixtures can possess quite varying molecular weights, polarities, and volatilities^40^. Significant amounts of impurities can occasionally be found in purportedly pure pigments^41^.

**Fig. 1:**
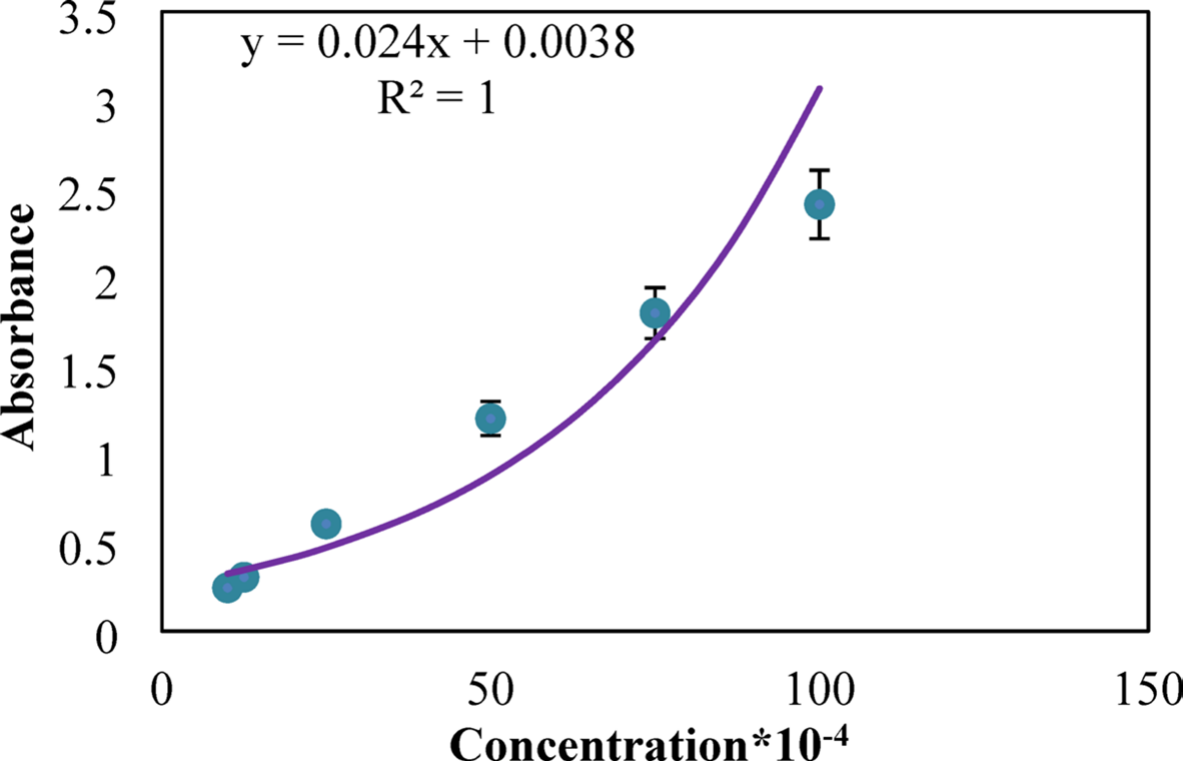
Measurement of molecular extinction coefficient of AB113 dye.

Acid Blue 113 (AB113), also called Neural Blue 5R, has an extinction coefficient (ε) of ≥ 18,000^42^. The commercial sample of AB113 which were used in the studies contain the dye content which was one of the samples was ascertained using the following method: Six different concentrations (1.00 × 10^−4^; 1.25 × 10^−4^; 2.50 × 10^−4^; 5.00 × 10^−4^; 7.50 × 10^−4^and 10.00 × 10^−4^) distilled water was used as a reference when measuring the absorbance at 566 nm (Fig. 1). For the latter, the commercial sample’s εAB113 was intended using the mean of six values as presented in Eq. 1.

1 $$\begin{gathered} \varepsilon_{{{\mathrm{AB113}}}} = \varepsilon_{{1}} + \varepsilon_{{2}} + \varepsilon_{{3}} + \varepsilon_{{4}} + \varepsilon_{{5}} + \varepsilon_{{6}} {/6} = {245}0 + {244}0 \hfill \\ \quad \quad \quad \quad\; + {2424} + {24}0{2} + {2396} + {2411/6} = {2421} \hfill \\ \end{gathered}$$

The commercial grade AB113’s purity was computed using the determined ε value.

### Preparation of the solutions for the application to textile industrial effluent [TIE]

#### Textile industrial effluent

A nearby textile industry that operates two shifts provided effluent samples. The TIE samples that were randomised were placed in 10 L polyethene containers at the dead end of the pipe, where the effluent is injected into the treatment plant. The three samples, taken consecutively over the three shifts of work, were used across the three workdays. All effluent samples from the textile industries were placed in a 100-L barrel and vigorously stirred by hand to ensure a uniform concentration. The solution obtained was used as the TIE control sample for analysis. A typical procedure was employed to collect, process, and preserve effluent samples^43^.

#### Dyeing the Acid Blue 113 dye in water

Two grams of AB113 dye were added to a 2-L standard flask. Distilled water was used, and the solution concentration varied by adding a dye. To achieve a uniform solution, the solution obtained (Solution 1) was agitated.

#### Preparation of Acid Blue 113 dye in textile industrial effluent

Two grams of AB113 dye were added to a 2-L standard flask. The remaining solution was added up to TIE, and the dye was dissolved. Following this, the solution obtained (Solution 2) was stirred until evenly concentrated (Solution 2).

### Procedures

#### Absorbance measurement of stock solution

The absorbance of the filtrate was read at a scale of maximum absorbance of 3.0, after the filtration of an aliquot solution of TIE at the Buchner funnel device using the No 42 Whatman filter paper on UV-Vis Spectrophotometer (Perkin Elmer-Lambda 25, USA). All the absorbances were analyzed in this range. Absorbance, on the other hand, was also estimated using the solution concentration by multiplying the concentrations of the resulting solution that was calculated by making a suitable dilution by the factor of dilution to be determined.

### Experimentation using the proposed method on textile industrial effluent [TIE]

*First step*: A one-litre conical flask was taken and filled with 500 mL of Solution 1. Once 0.500 g of MNC was added to the conical flask, the latter was stirred on a magnetic stirrer at 700 rpm. Agitation for 15 min was stopped, and the solution was collected using a Buchner funnel with No. 42 Whatman filter papers. Regeneration commonly involves repeating the filtration, and the absorbance is measured when the filtrate is no longer visible.

*Second step*: We added another volume of 0.250 g of MNC to the conical flask used to prepare the filtration, as we did in step 1. The mixture was immediately placed in a magnetic stirrer at 700 rpm for 15 min. Agitation ceased after 15 min, and the solution was filtered through a Buchner funnel using No. 42 Whatman filter paper. If the filtrate does not appear clear, the procedure is repeated, and the absorbance is noted.

*Third step*: Upon addition of the third loading, 0.250 g MNC, to the same conical flask containing the filtration solution from Step 2, stirring was performed for 15 min using a magnetic stirrer at an approximate stirring speed of 700 rpm. The agitation was stopped after 15 min, and the Buchner funnel-based equipment was used to filter the solution through No. 42 Whatman filter paper. Regeneration commonly involves repeating the filtration, and the absorbance is measured when the filtrate is no longer visible.

**Fig. 2:**
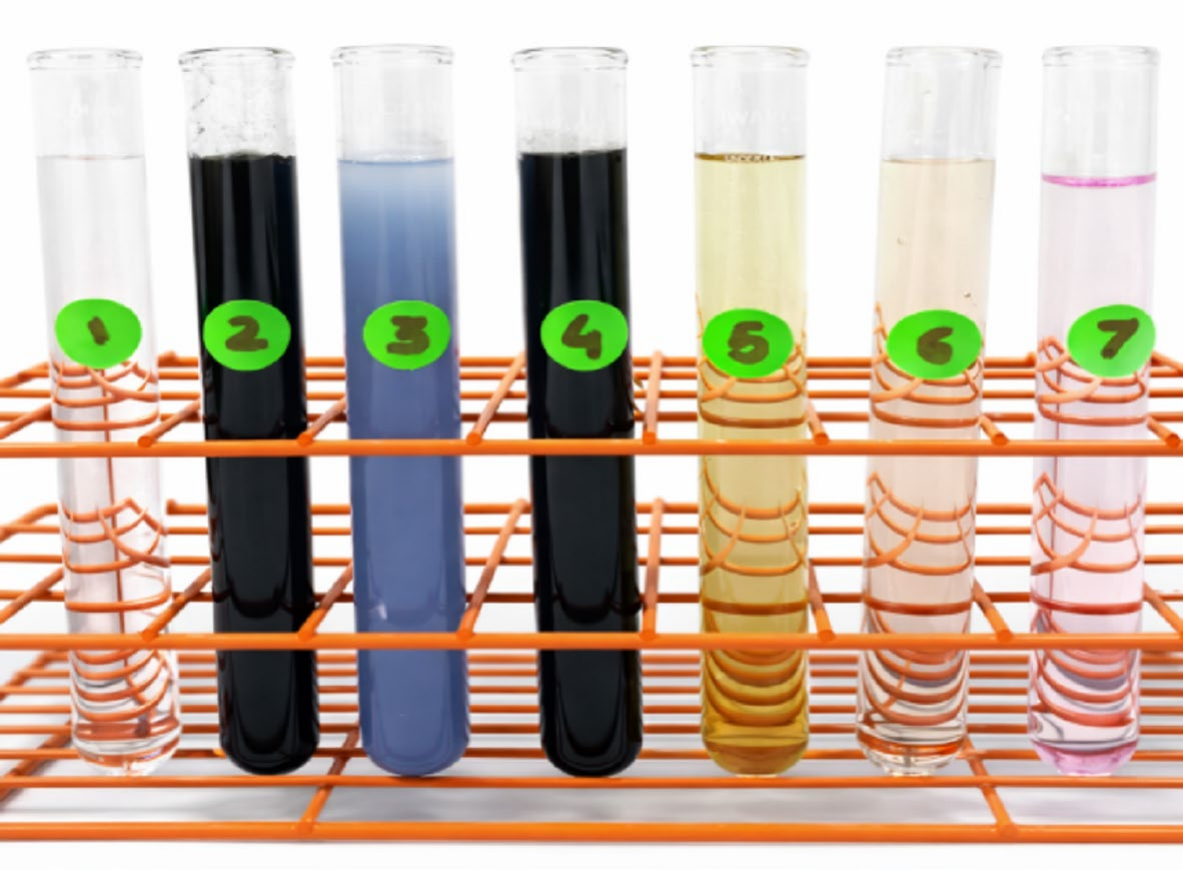
Before and after adsorption solution colours44: 1. Distilled water; 2. AB113 dye in distilled water; 3. TIE; 4. AB113 dye in TIE; 5. Filtrate after adsorption of dye on MNC after 15 min; 6. 30 min; 7. 45 min.

*Fourth step*: Solution 2 was used to repeat Steps 1 through 3. The filtrate solutions are shown in Fig. 2 after the components of Solution 2 have adsorbed on MNC.

### Adsorbent

MNC was sourced from a local supplier to ensure the sample resembles those used in real-world applications. The MNC was dehydrated in a hot oven for around 12 h at 60 °C and the dehydrated sample was crushed and sieved in case there were any agglomerates identified. Additional research was conducted using the sieved powder.

### Surface description

The electron microscopes examined were a LEO 435 VP, a Japanese-manufactured instrument, used to examine the surface morphology of MNC. To determine the presence of functional groups in the adsorbent, Fourier transform infrared spectroscopy (FTIR) with an InterSpec 2020 FTIR spectrometer manufactured by Spectro Lab, UK, was used. This technique was used to obtain infrared spectra of the MNCs loaded with AB113 and the control sample (MNCs prior to loading with AB113). The flasks containing the MNC adsorbent (10 150 mL Erlenmeyer flasks) were weighed accurately (0.05 g) and then correctly labelled. 0.1 M of KNO_3_ solution (25.0 mL) of the desired starting pH was added to each of the flasks and shaken accordingly under ideal conditions of contact time. Working dilute solutions of 0.1 M NaOH and HCl were used to vary the starting pH to the required pH. The solutions were then placed in an orbital shaker at room temperature and 250 rpm for 24 h. They were shaken and filtered into sterile glass vials labelled with a metal mesh. The pH of each filtrate was measured by using a pH meter. The change in pH was calculated, and the resulting graph was plotted against the initial pH to determine the point-of-zero-charge (pH_pzc).

### Batch adsorption experiment

Different setups were used to conduct the trials in batch mode. Each experiment was to be conducted by preparing all 250 ml flasks with 50 ml of an aqueous AB113 dye solution at a concentration of 150 mg L^−1^, and each flask contained 50 mg of MNC. Three hours passed, during which the flasks were shaken at 165 rotations/min with a temperature-controlled shaker. To explore the implications of the article, the following variables were tested: temperature (303 K, 313 K and 323 K) with dye concentrations of 75, 150 and 300 mg L^−1^, dosage of MNC (varying between 0.025 and 0.300 g L^−1^) and the amount of the AB113 dye (25–1000 mg L^−1^). The centrifuge was used to wash off the remaining particulate matter for another 5 min at 3000 rpm. The remaining filtrate absorbance was measured at 566 nm using a UV–Vis Spectrophotometer (Perkin Elmer Lambda 25, USA). Experiments with the adsorbent in distilled water and in a dye solution without the adsorbent were conducted to establish controls.

To calculate the optimum MNC concentration, measured as the mass of dye, a series of MNC concentration gradients (0.500–6.000 g L^−1^) was tested until a steady state was attained. Additionally, dye-adsorption characteristics were studied using an orbital shaker, agitating 50 mg of MNC in 50 ml of 150 mg L^−1^ dye solutions at pH 2.12. Although agitation was maintained for 180 min, equilibrium was reached after 140–150 min at a constant high agitation velocity (165 rpm). The final dye concentration was measured at 566 nm using a double-beam UV–Vis spectrophotometer. After pH correction with dilute HCl or NaOH, pH was measured using a pH meter (Systronics 802, India). Each adsorption experiment was repeated thrice, and the mean of the three replicates was reported as the result.

### Statistical optimization of process parameters

Two levels of standard experimental design were created using five components. These factors include contact time (0–180 min; A), temperature (27–50 °C; B), initial concentration of the dye (25–1000 mgL^−1^; C), dose of the adsorbent used (0.500–6.000 g L^−1^; D) and initial pH (2–12; E) because they influence final adsorption capacity and the adsorption process. The dependent variable that required these independent variables to be controlled was adsorption capacity, measured under fixed orbital shaking at 165 rpm. In the ANOVA, general quadratic relapse equivalence was found. Surface plots and contour plots were also generated, which are used symbolically to illustrate the effects of one at a time and their combinations on the adsorption capacity^44^.

## Results and discussion

### Characterization of adsorbent surface

**Fig. 3:**
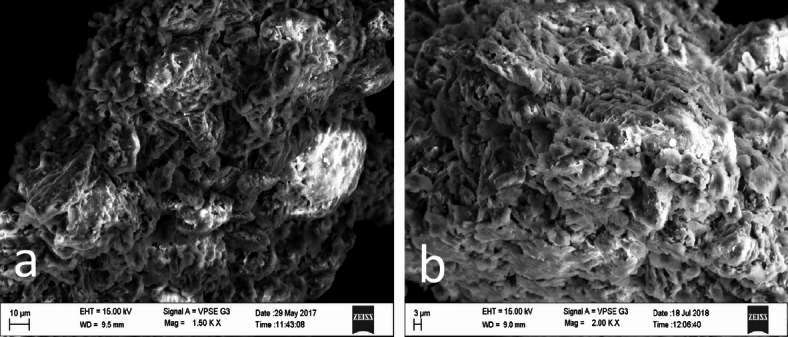
SEM image of MNC and AB113 dye adsorbed MNC.

**Fig. 4:**
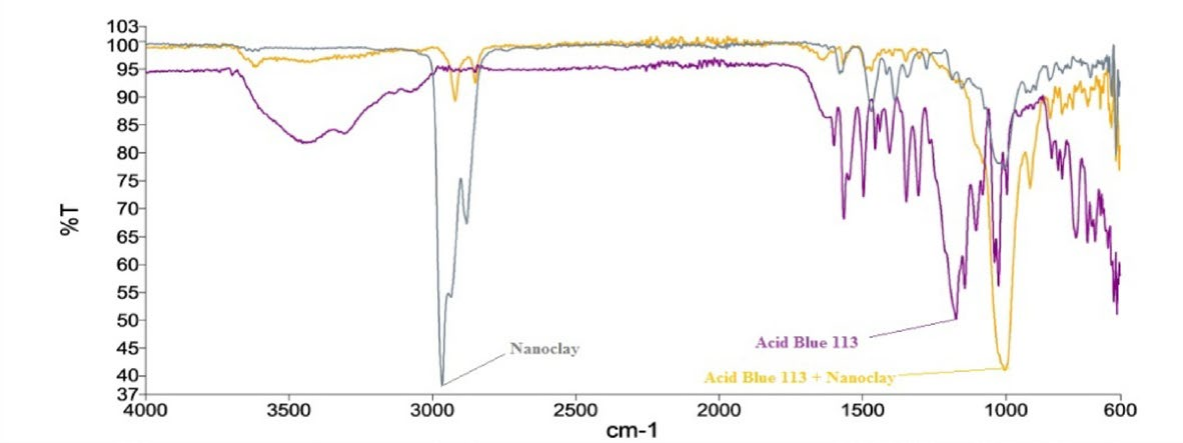
Adsorption FTIR spectrum analysis.

The porous structure was shown by SEM surface characterisation of MNC (Fig. 3). After adsorption, the particle was covered by a thin layer of adsorbate that filled parts of the pores on AB113 (Fig. 3). The band width, as shown in the IR spectrum 3353–3454 cm^−1^ in Fig. 4, is due to the adsorbed water molecule and hydroxyl groups, as explained by MNC IR spectra (Fig. 4). The C–H stretch gives a sharp band at 2852 cm^−1^, and the C–O stretch is represented as a band at 1598 cm^−1^.

**Fig. 5:**
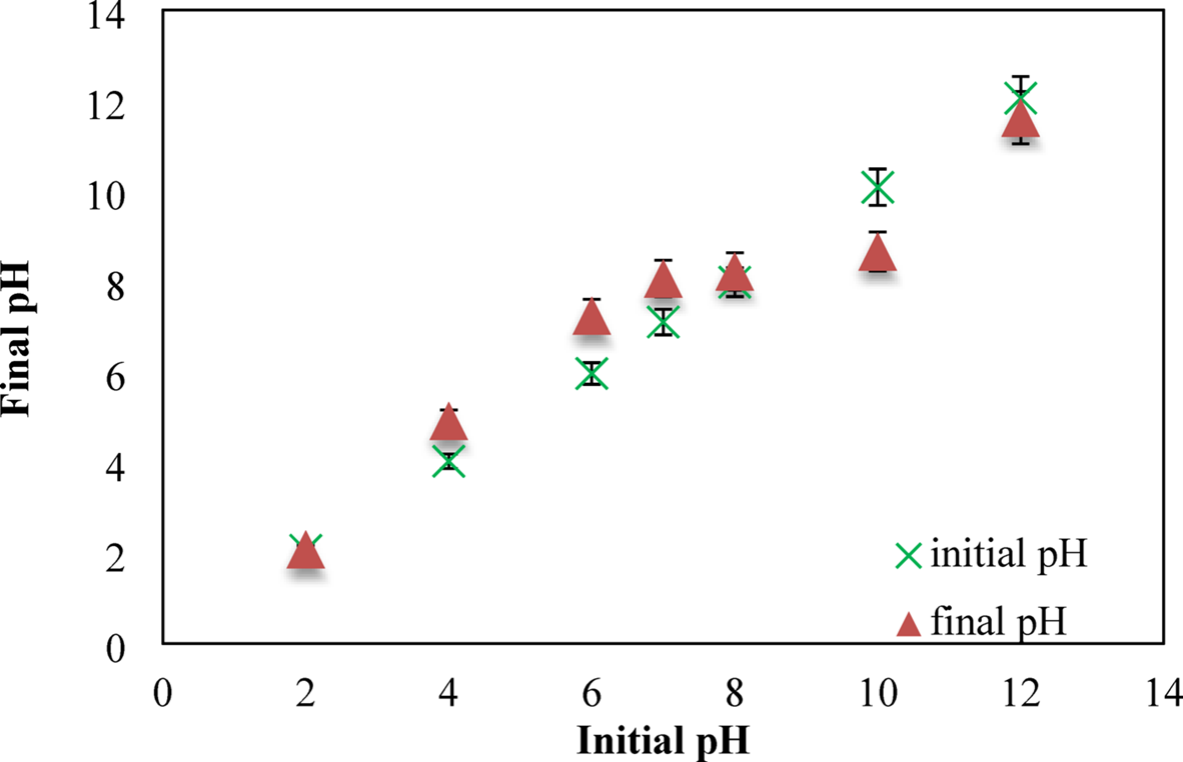
Point-of-zero-charge of MNC.

More so, C–O–C stretching results in bands at 1418, 1404, 1355, 1158, and 1091 cm^−1^. In the spectra obtained in the IR region of 3300–3500 cm^−1^, a 3353–3454 cm^−1^ band, large peaks were found to denote the hydroxyl groups of the MNC, and they are due to the broadening of the -NH_2_ group in the AB113 dye that has disappeared after the dye has adsorbed on the MNC. This finding is in favour of the existence of hydrogen bonding between the -NH_2_ and the hydroxyl groups. Additionally, AB113 dye exhibits significant adsorption on MNC, as evidenced by a prominent peak in the N–N stretching region at 1598 cm^−1^, which vanishes. Lastly, it deduces that the absence of IR absorption frequencies indicates that AB113 dye has mostly adsorbed onto MNC. The point-of-zero-charge, obtained at the intersection of two plots (Fig. 5), confirms that there are no charges on the adsorbent surface at pH 8.22.

### Batch adsorption studies

#### Effect of pH and initial dye concentration

**Fig. 6:**
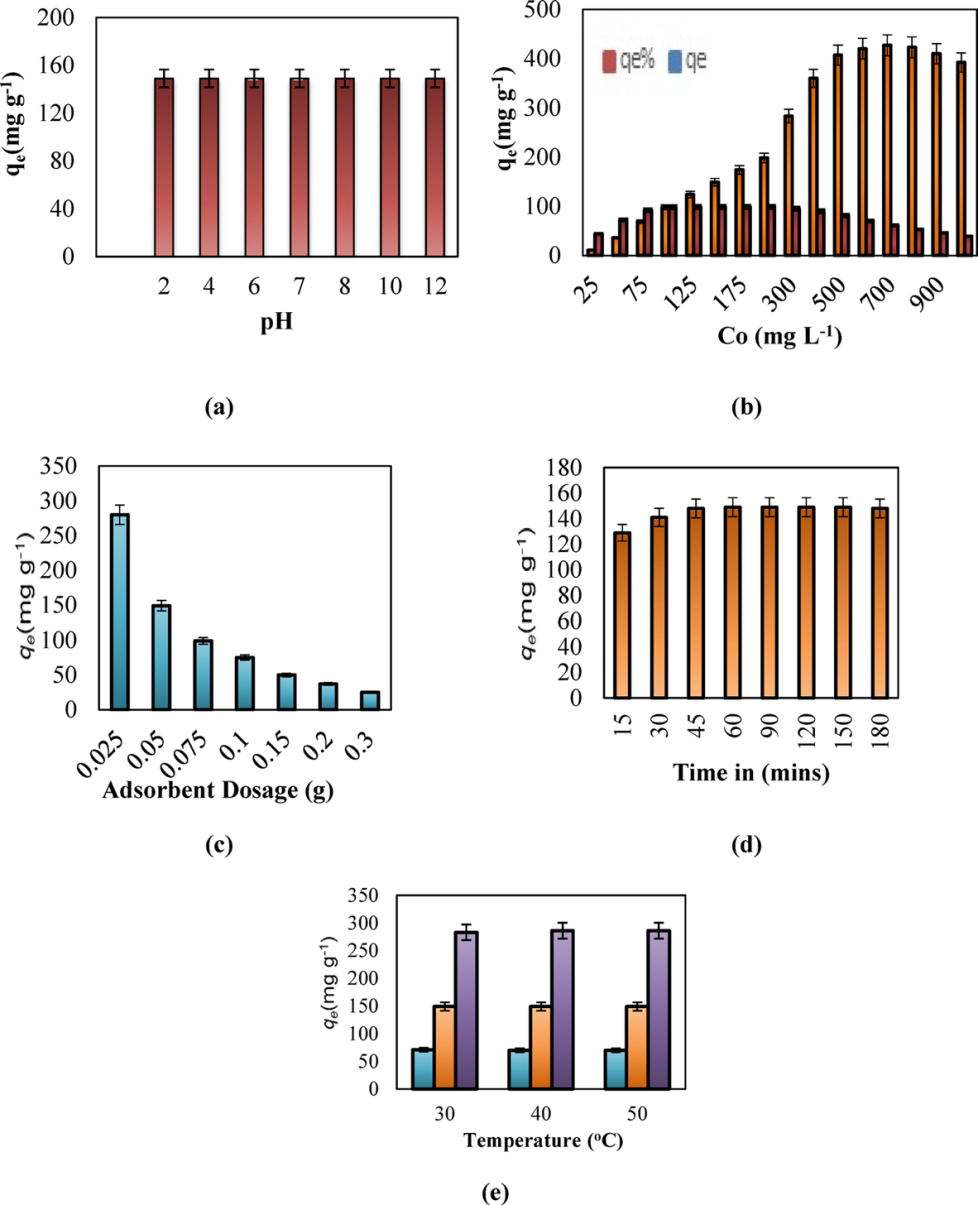
Parametric effect on adsorption of (**a**) solution pH, (**b**) initial dye concentration, (**c**) adsorbent dosage, (**d**) contact time and (**e**) temperature.

To achieve the highest level of adsorption, the optimal conditions for each parameter should be determined. pH has been the most widely considered parameter because it accounts for the adsorption process, which influences the ionic form of the dye molecules in solution and the surface characteristics of the adsorbent, thereby affecting the adsorption capacity, among other factors. The maximum amount of AB113 dye was removed by MNC at pH 2.0, 4.0, 6.0, 7.0, 8.0, 10.0, and 12.0 (*q*_*e*_ = 149.00 mg g^−1^) at an initial concentration of 150 mg L^−1^ (Fig. 6). The *q*_*e*_ value increases when the initial dye concentration increases from 25 to 200 mg L^−1^, as shown in Fig. 6. As concentration increased, the percentage *q*_*e*_ value rose as well, peaking in the 125–200 mg L^−1^ concentration range. The % *q*_*e*_ value generally remains constant after that.

The negligible influence of pH (2–12) on the adsorption of AB113 onto MNC suggests that the adsorption process is not predominantly governed by electrostatic interactions. Montmorillonite possesses a permanent negative structural charge arising from isomorphic substitution within its lattice, which remains largely unaffected by pH variations. Although edge sites exhibit pH-dependent behaviour (pHpzc ~ 2–3), their contribution is minimal compared to the dominant basal surface charge. Therefore, changes in solution pH do not significantly alter the overall adsorption capacity.

The adsorption of AB113 is more likely controlled by non-electrostatic interactions, including hydrogen bonding between dye functional groups and surface hydroxyls, van der Waals forces, and possible π–π interactions between the aromatic rings of the dye and the siloxane surface. Additionally, cation bridging via exchangeable interlayer cations (e.g., Na^+^, Ca^2+^) may facilitate dye adsorption. The large molecular size and structural stability of AB113 further reduce its sensitivity to protonation–deprotonation effects across the studied pH range. Collectively, these factors account for the observed pH-independent adsorption behavior.

Influence of pH (2–12) on the adsorption of AB113 onto MNC suggests that the adsorption process is not predominantly governed by electrostatic interactions. Montmorillonite possesses a permanent negative structural charge arising from isomorphic substitution within its lattice, which remains largely unaffected by pH variations. Although edge sites exhibit pH-dependent behaviour (pHpzc ~ 2–3), their contribution is minimal compared to the dominant basal surface charge. Therefore, changes in solution pH do not significantly alter the overall adsorption capacity.

The adsorption of AB113 is more likely controlled by non-electrostatic interactions, including hydrogen bonding between dye functional groups and surface hydroxyls, van der Waals forces, and possible π–π interactions between the aromatic rings of the dye and the siloxane surface. Additionally, cation bridging via exchangeable interlayer cations (e.g., Na^+^, Ca^2+^) may facilitate dye adsorption. The large molecular size and structural stability of AB113 further reduce its sensitivity to protonation–deprotonation effects across the studied pH range. Collectively, these factors account for the observed pH-independent adsorption behaviour.

#### Effect of adsorbent dosage

Adsorbent dose also influences the adsorption process rather significantly as it figures out the extent of adsorption capacity of a given initial concentration of the adsorbate in the condition of operation. This was an experiment in which the influence of the adsorbent dosage of the AB113 dye was surveyed over the following interval: 0.500–6.000 g L^−1^. It emerged that the percentage adsorption of the AB113 dye increased with increasing adsorbent amount. The adsorbent used is higher because the AB113 dye transfers to MNC more effectively. The adsorbate yield was unaffected by any further increases in adsorbent dosage beyond the limit. Adsorbent dose also significantly influences the adsorption process, as it determines the adsorbate concentration at a given initial concentration under the conditions of operation. This was an experiment in which the influence of the adsorbent dosage of the AB113 dye was surveyed over the following interval: 0.500–6.000 g L^−1^. It emerged that the percentage adsorption of the AB113 dye increased with increasing adsorbent amount. The adsorbent used is higher as the AB113 dye transfer to MNC is better (Fig. 6).

#### Effect of contact time on dye adsorption

The impact of contact duration and AB113 dye absorption on MNC was observed for 15, 30, 45, 60, 90, 120, 150, and 180 min, and the first 15 min of the experiment demonstrated rapid adsorption, with 86% of the total adsorption occurring. However, as seen in (Fig. 6), the adsorption rate increased steadily throughout the course of the contact period until it reached equilibrium after 90 min. Dye molecules penetrate further and at higher energy as they accumulate over longer contact times into the adsorbent structure. The aggregation removes the impact of contact time, as it involves blocking the mesopores and introducing resistance to the diffusion of aggregated dye molecules within the adsorbents.

#### Effect of temperature

The effect of temperature on the adsorption of AB113 onto MNC was evaluated over the range of 30–50 °C at different initial dye concentrations. As shown in Fig. 6e, the adsorption capacity (*q*_*e*_) remains nearly constant across the investigated temperature range, indicating that temperature has a negligible influence on the adsorption process. This behaviour suggests that the adsorption is not strongly thermally activated and is likely governed by physisorption mechanisms, including van der Waals interactions, hydrogen bonding, and diffusion-controlled processes. The minimal temperature dependence indicates that the interaction between AB113 and MNC remains stable over the studied range, highlighting the system’s robustness for practical applications without strict temperature control^45^. Table 1 summarises key parameters, including maximum adsorption capacity (*q*_*e*_), operating conditions, and distinguishing features of various adsorbents reported in the literature.

**Table 1:** Comparative performance analysis with reported adsorbents.

Adsorbent	*q*_*e*_ (mg g^−1^)	Conditions	Key limitations	Reference
Activated carbon (waste tyre)	~ 200–300	Narrow pH range, High cost	Expensive, Regeneration issues	^20^
Red mud	~ 100–150	pH dependent	Secondary waste generation	^22^
Fly ash	~ 80–120	Moderate conditions	Lower efficiency	^21^
Biosorbents (agro-waste)	~ 50–150	Variable	Low stability, Poor reusability	^1^
Nano zero-valent iron	~ 150–250	Controlled conditions	Cost, Aggregation	^19^
MNC	149	pH 2–12, 30–50 °C	Minimal limitations	Present work

### Adsorption isotherms

These are the isotherm properties that contribute significantly to the interpretation of the adsorbate’s interactions with the adsorbent surface. The process of the AB113 dye onto MNC has been explained with adsorption isotherms. The two widely recognised models for determining adsorption capacity or fitting experimental data include the Freundlich and Langmuir isotherms. The Langmuir model assumes a monolayer coverage of the surface with an infinite number of adsorption sites of the same type, each having the same strength^46^. The equilibrium was determined using the original AB113 dye concentration of 25–1000 mg L^−1^. The measured value of *Q*_*m*_, 375.15 mg g^−1^, for this isotherm is excessively large relative to the experimental *q*_*e*_ value of 149.00 mg g^−1^. Nevertheless, the *R*^2^ of 0.84 indicates that this isotherm performs well in fitting the trial statistics. The calculated *R*_*L*_ values, ranging from 0.004 to 0.021, indicated that the AB113 dye was adsorbed sympathetically onto MNC. The *R*_*L*_ value decreases with increasing initial concentration, indicating that higher concentrations are more favourable for adsorption^47^. *Q*_*m*_ (375.15 mg g^−1^) and *q*_*e*_ (149.00 mg g^−1^) differed significantly; the authors have considered different adsorption isotherm models. The Freundlich isotherm is an empirical equation that describes adsorption on heterogeneous surfaces^48^. The Langmuir isotherm has been extended to the Jovanovic isotherm^49^. Physisorption prefers the typical Langmuir isotherm with *n*_*F*_ and 1/*n*_*F*_ values of 4.161 and 0.240, respectively.

**Fig. 7:**
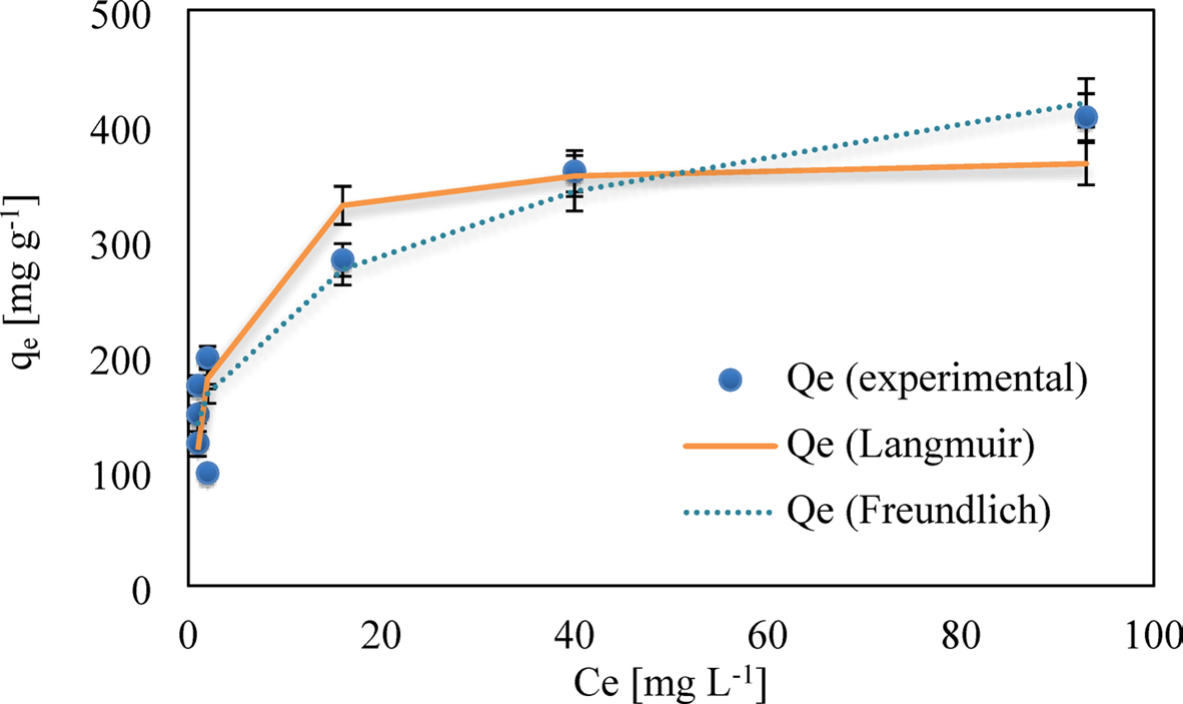
Experimental and isotherm adsorption models.

**Fig. 8:**
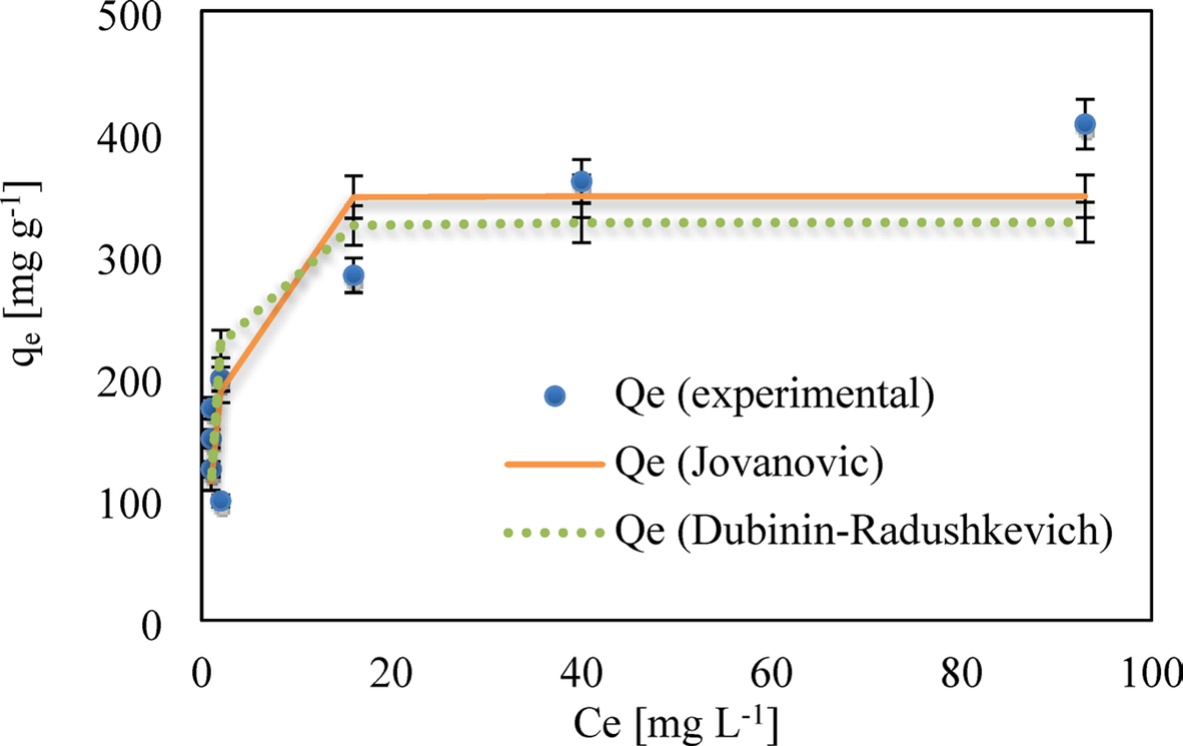
Experimental and isotherm adsorption models.

**Fig. 9:**
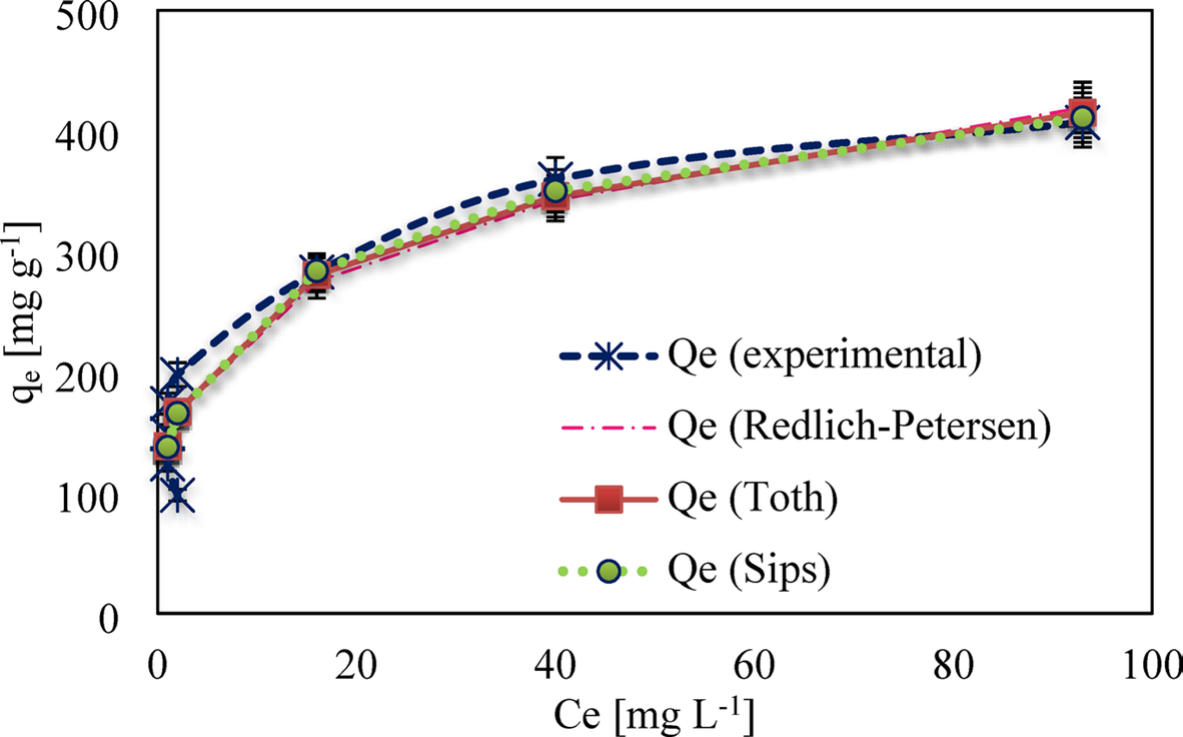
Experimental and isotherm adsorption models.

It may be concluded that AB113 dye adsorption onto MNC is advantageous under the experimental conditions and that physisorption is the dominant process. Instead, the authors examined an extended model to fit the data, as the Langmuir and Freundlich models could not determine whether the system was homogeneous or heterogeneous (Fig. 7). The first is the Dubinin-Radushkevich isotherm^50^ that was originally introduced to the adsorption process through the pore-filling model. The Dubinin-Radushkevich isotherm showed *q*_*s*_ = 326.58 mg g^−1^, which is greater than the experimental value. The fact that fitting the Dubinin-Radushkevich isotherm to the experimental data yields a correlation coefficient (*R*^2^) of 0.98 indicates that the process is linear and tends to describe the data somewhat better than the Langmuir isotherm. The Jovanovic isotherm yielded a large *Q*_*m*_ value of 347.85 mg g^−1^, compared to the experimental value of *q*_*e*_ 149.00 mg g^−1^, due to the similarity of two parameters, namely, *χ*^2^ and *R*^2^ (Fig. 8). The fact that the dye AB113 interacts with MNC in a linear, positive, and physical manner was confirmed by Langmuir and Freundlich; their findings are reported in Tables 2 and 3. For academic use, the other 6 three-parameter isotherm models were also explored, including Toth, Sips, Radke-Prausnitz, Redlich-Peterson, Vieth-Sladek, and Brouers-Sotolongo. The Toth isotherm is an empirical model that is considered to fit the Langmuir isotherm better and to represent heterogeneous adsorption systems^51^. The *Q*_*m*_ value of 9067.60 mg g^−1^, as found in Table 4 and Fig. 9, is larger than the *q*_*e*_ value of 149.00 mg g^−1^ that was experimentally determined and the Langmuir isotherm value of 375.15 mg g^−1^.

**Table 2:** Isotherm parameters calculated and applied to two limitations.

Langmuir		Freundlich		Jovanovic		Dubinin-Radushkevich
*Q*_*m*_	375.15	*K*_*F*_	141.17	*Q*_*m*_	347.85	*Q*_*s*_ 326.58
*K*_*S*_	0.461	*n*_*F*_	4.161	*K*_*J*_	0.388	*K*_*ad*_ 3.49E-07

**Table 3:** Isotherm parameters calculated and applied to three limitations.

Redlich-Peterson		Toth		Radke-Prausnitz		Sips		Vieth-Sladek		Brouers-Sotolongo	
*A*_*RP*_	34,136	*Q*_*m*_	9067.60	*Q*_*m*_	21.6	*Q*_*m*_	529.4	*Q*_*m*_	281.5	*Q*_*m*_	693.3
*B*_*RP*_	241.006	*n*_*T0*_	0.077	*K*_*rp*_	2467.548	*K*_*s*_	0.007	*K*_*VS*_	1.498	*K*_*BS*_	0.222
*g*	0.760	*b*_*T0*_	0.379	*m*_*r*_	0.759	*m*_*s*_	0.27	*β*_*VS*_	0.83	*α*	0.31

**Table 4:** Statistical terms for the model.

Isothrms	Langmuir	Freundlich	Jovanovic	Dubinin-Radushkevich	Redlich-Peterson	Toth	Radke-Prausnitz	Sips	Vieth-Sladek	Brouers-Sotolongo
SSE	14,999.6	7665.7	21,309.5	31,963.5	7666.6	7575.9	7665.9	7377.3	10,138.9	7499.5
*χ*^2^	106.518	63.617	138.989	230.221	63.679	64.000	63.614	62.549	85.456	63.531
*R*^2^	0.84	0.91	0.77	0.65	0.91	0.91	0.91	0.92	0.89	0.92

The Sips isotherm represents the use of the Freundlich and Langmuir isotherms combined^52^. The Freundlich equation is only applicable to replace the Sips equation at low adsorbate concentrations. When the adsorbate is in high concentration, the formula provides the normal monolayer adsorption capacity of the Langmuir isotherm. The *Q*_*m*_ = 529.40 mg g^−1^ solution obtained from the Sips isotherm shows a small difference relative to the experimental value, as does the Langmuir isotherm. The values of lower SSE (7377.3), *χ*^2^ (62.549), and higher *R*^2^ (0.92) are quite close to those of the Langmuir model and give a fair fit to Experimental results. For the isotherm model to fit optimally, a well-balanced *Q*_*m*_ prediction is required, and the estimated value should closely mirror the experimental value. According to Fig. 9, a smaller *χ*^2^ value denotes greater similarity to the experimental data. The projected *Q*_*m*_ value of 21.60 mg g^−1^ reported by the Radke- Prausnitz isotherm was however significantly lower than that of the experimentally determined *Q*_*m*_ value^53^. The attained g-value is 0.76, which inflates that the Langmuir isotherm is an adsorption direction^54^.

**Fig. 10:**
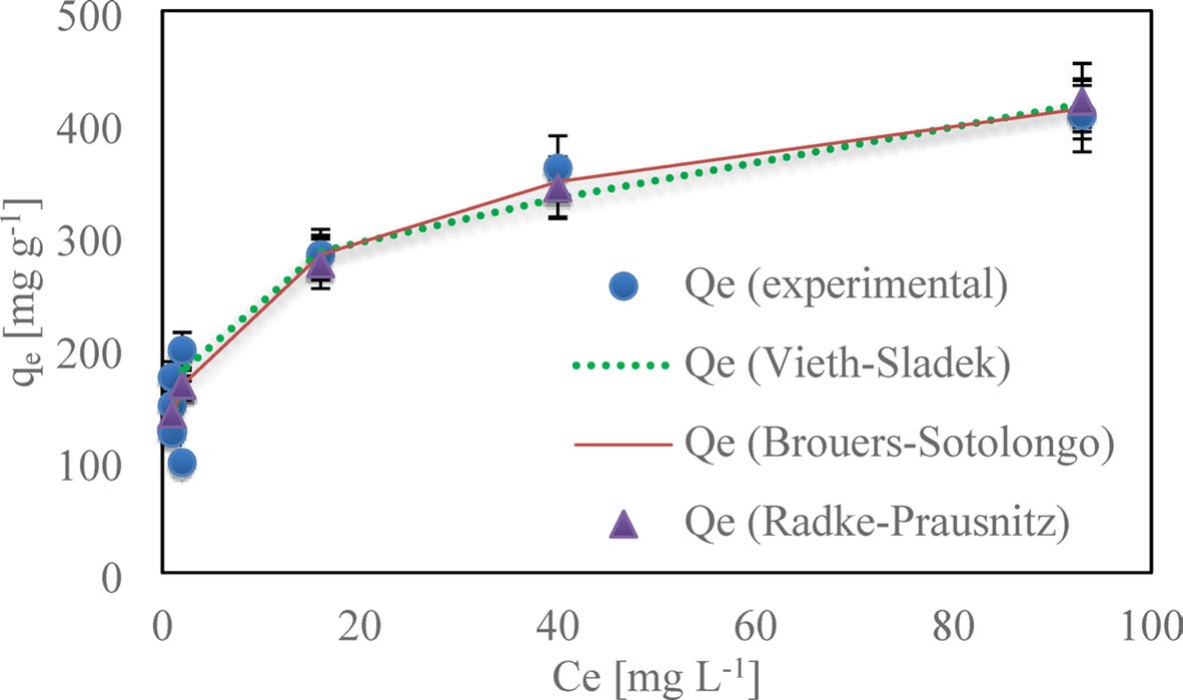
Experimental and isotherm adsorption models.

The ViethSladek isotherm^55^ was originally applied to solutes that were adsorbed following a certain isotherm that reduced a non-linear portion (Langmuir equation) and a linear portion (Henry’s law) of the solute was adsorbed. The nonlinear component demonstrates solute adhesion to specific areas of porous adsorbent surfaces. Conversely, the physical relationship between the linear component and solute dispersion on segments of the amorphous components of the adsorbent polymers is involved. When the present model is contrasted with the other models investigated, the anticipated *Q*_*m*_ value of 281.50 mg g^−1^ is calculated. The Vieth-Sladek isotherm model, which forecasts the diffusion rate of solid materials through transient adsorption, could be compared with the Brouers-Sotolongo isotherm^56^, as it yields a *Q*_*m*_ value of 693.30 mg g^−1^, which is higher than the experimental result of 149.00 mg g^−1^. However, the experimental data is accurately predicted by this isotherm model with an *R*^2^ value of 0.92 (Fig. 10). *R*^2^ values are limited to linear models, which are used to understand the adsorption mechanism, whereas higher-order equations are not. As a result, it cannot be used on its own to verify that the data fit is accurate. Values of *χ*^2^ provide a more telling indication of whether the experimental data and the model are similar, and vice versa. All nine isotherm models provided values of the critical parameters (*Q*_*m*_, *χ*^2^ and *R*^2^). The mismatch between the parameter values of all considered models and the experimental values (*q*_*e*_) will arouse interest in the scientific community, especially among researchers in mathematical modelling. The authors could develop new models to have a better perception of the adsorption process in the system AB113-MNC.

### Adsorption kinetics

In determining potential control levels for adsorption, kinetic models are appropriate^29^. In the kinetic analysis, AB113 dye was used at concentrations of 75, 150, and 300 ppm. The alteration in the adsorption rate is disclosed from the results of kinetic studies at three distinct temperatures (303 K, 313 K, and 323 K). Kinetic adsorption data were analysed nonlinearly using pseudo-first-order^57^, pseudo-second-order^58^, and the Weber-Morris model of intraparticle diffusion^59^ in MS Excel 2010. The Dumwald-Wagner model^60^ and the Film Diffusion model^61^. The estimated parameters have been presented in Tables 5 and 6.

**Table 5:** Theoretically and experimentally determined models of the kinetics of absorption.

Initial concentration (ppm)	Temp (K)	*q*_*eexpt*_ (mg g^−1^)	Pseudo-first-order				Pseudo-second-order			
			*q*_*epred*_ (mg g^−1^)	*k*_1_	*R*^2^	*χ*^2^	*q*_*epred*_ (mg g^−1^)	*k*_2_	*R*^2^	*χ*^2^
75	303	71	67.58	2.59E-01	0.52	0.26	70.07	1.11E-02	0.82	0.31
	313	70	67.36	2.64E-01	0.56	0.20	69.65	1.21E-02	0.86	0.07
	323	70	67.36	2.64E-01	0.56	0.20	69.65	1.21E-02	0.86	0.07
150	303	149	145.48	2.26E-01	0.88	0.16	151.55	4.21E-03	0.99	0.01
	313	149	145.72	1.46E+02	0.93	0.15	65.88	1.71E-03	0.76	1.65
	323	149	143.44	2.26E-01	0.63	0.67	150.40	3.80E-03	0.90	0.19
300	303	283	272.93	1.96E-01	0.94	0.28	287.80	1.63E-03	0.99	0.07
	313	286	277.44	1.99E-01	0.91	0.42	292.32	1.64E-03	1.00	0.01
	323	286	277.60	1.73E-01	0.93	0.47	296.89	1.20E-03	0.99	0.04

**Table 6:** Models of the parametric control of diffusion.

Initial concentration (ppm)	Temp (K)	Film diffusion model		Weber-Morris model		Dumwald-Wagner	
		*R*/(min^−1^)	*R*^2^	*k*_*ist*_ (mg g^−1^ s^−0.5^)	*R*^2^	*K* (min^−1^)	*R*^2^
75	303	0.0378	0.89	1.52	0.95	0.037	0.89
	313	0.0384	0.95	1.38	0.93	0.038	0.95
	323	0.0384	0.95	1.38	0.93	0.038	0.95
150	303	0.0552	0.99	3.49	0.89	0.054	0.97
	313	0.0578	0.99	4.09	0.86	0.057	0.99
	323	0.0604	0.97	4.15	0.99	0.059	0.99
300	303	0.0435	0.95	8.39	0.85	0.042	0.95
	313	0.0469	0.96	8.40	0.88	0.046	0.96
	323	0.0582	0.98	10.73	0.88	0.059	0.98

**Fig. 11:**
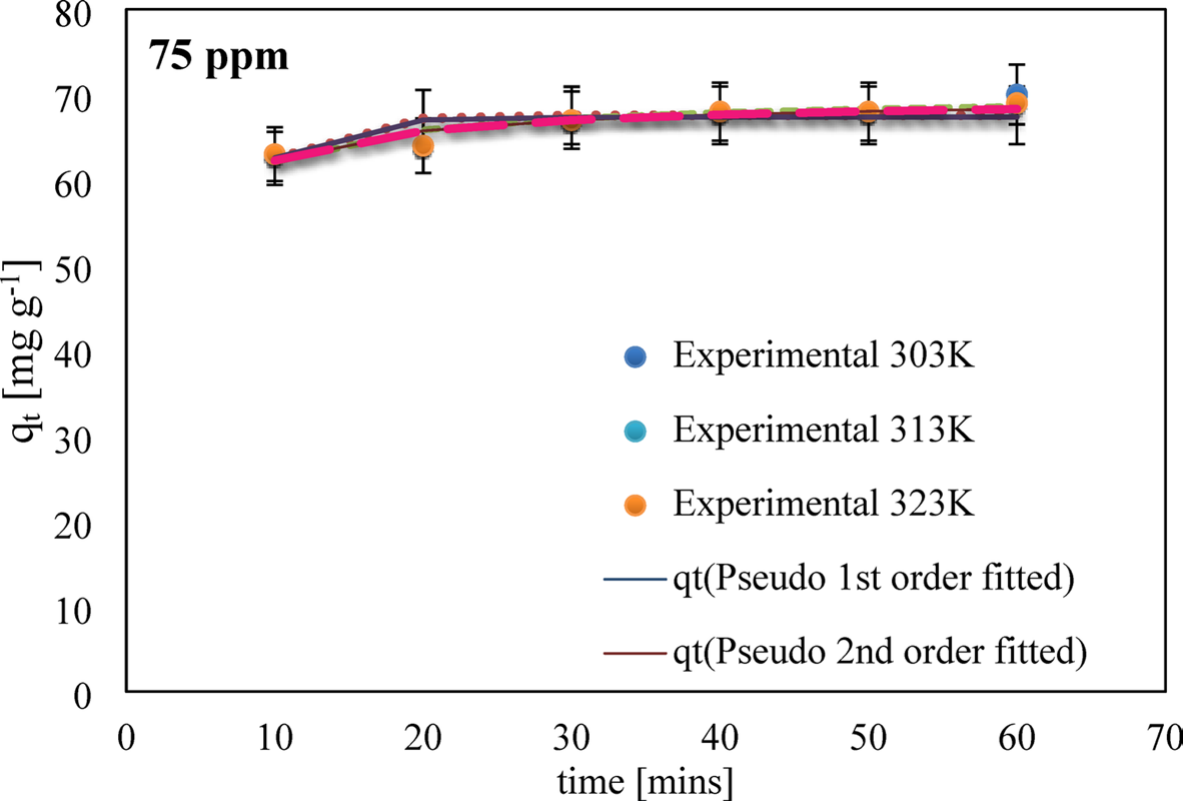
75 ppm preliminary concentration of AB113 dye on MNC system at dissimilar temperatures.

**Fig. 12:**
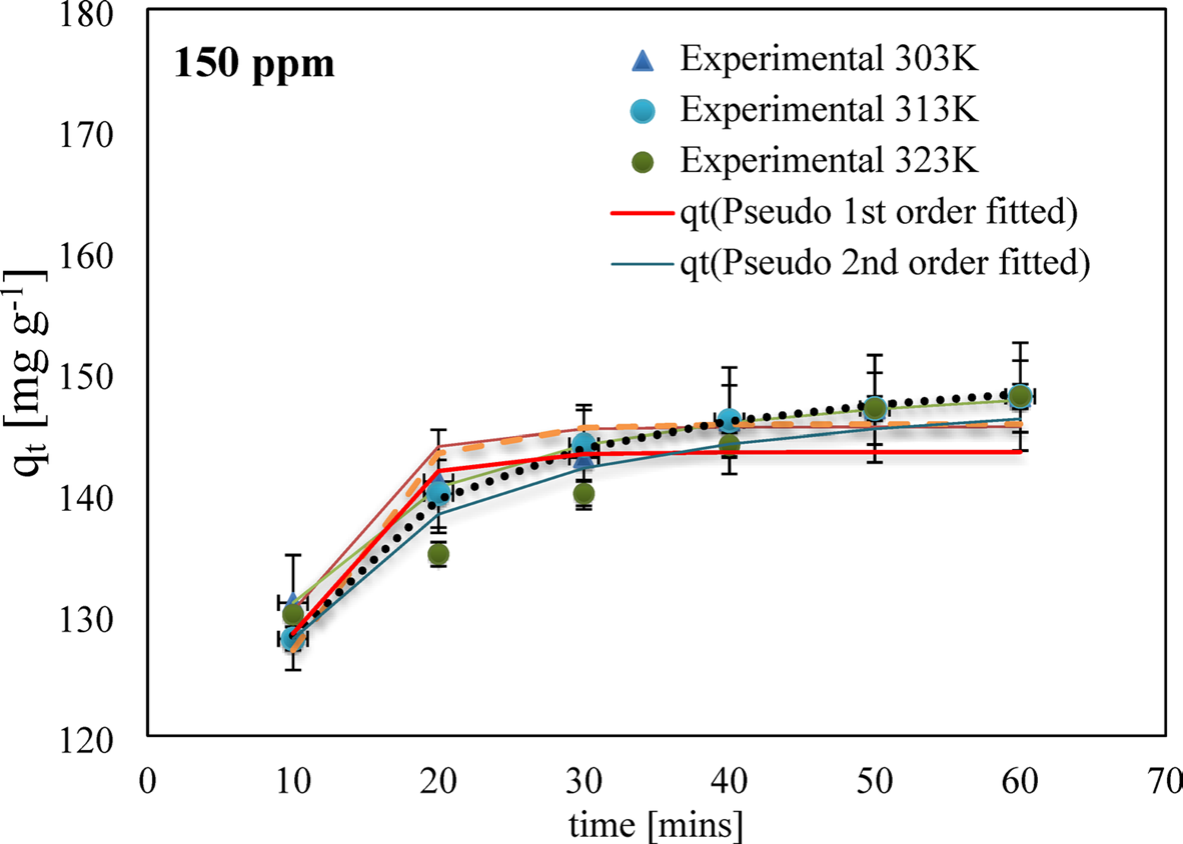
150 ppm preliminary concentration of AB113 dye on MNC system at dissimilar temperatures.

**Fig. 13:**
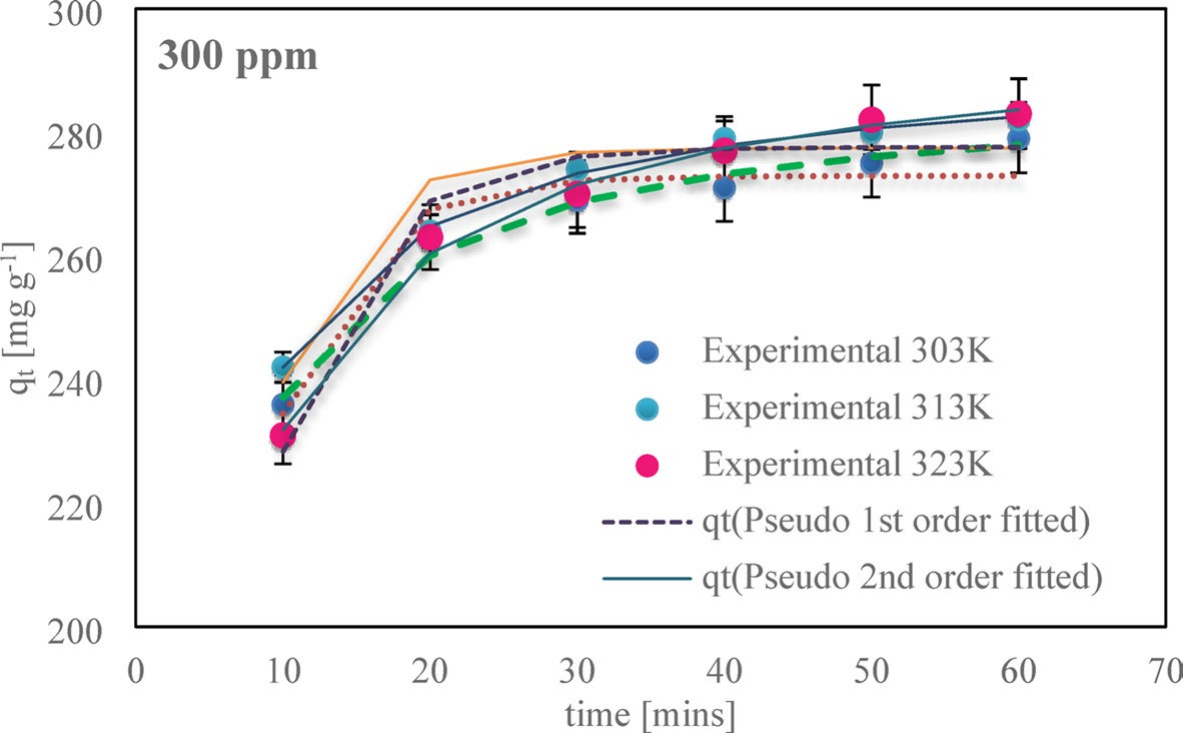
300 ppm preliminary concentration of AB113 dye on MNC system at dissimilar temperature

Based on the coefficients of the determination (*R*^2^) and the chi- squares (*χ*^2^) values, the pseudo- second- order model had an improved fit to the experimental results at different temperature levels of lower initial AB113 dye levels of 75, 150 and 300 ppm using the pseudo-second-order model related to the pseudo-first-order model (Figs. 11, 12 and 13). After the maximum adsorption, the high yield at the beginning of adsorption gradually declined and levelled off. The temperature rose, leading to an increment in adsorption capacity (*q*_*e*_). The outcomes affirm that there is no restriction on the rate due to the adsorption processes. The findings also show that the solute molecules moved from the bulk maximum phase towards the solid exterior and into the MNC pores during various adsorption processes.

**Fig. 14:**
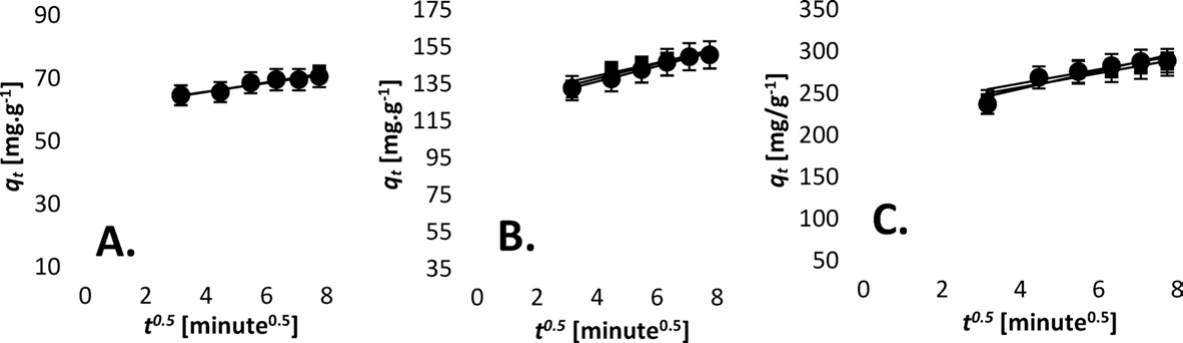
Dumwald-Wagner model with preliminary concentration of AB-113 dye: (**A**) 75 ppm, (**B**) 150 ppm, (**C**) 300 ppm.

**Fig. 15:**
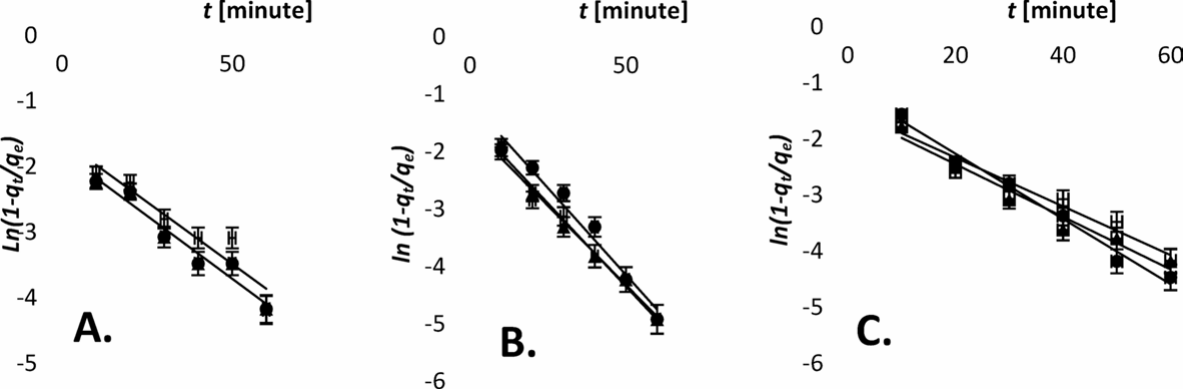
Kinetics data fitted to the Weber-Morris model with preliminary concentration of AB-113 dye: (**A**) 75 ppm, (**B**) 150 ppm, (**C**) 300 ppm.

The real *K* is calculated (Fig. 14) using the Dumwald-Wagner model, which accounts for diffusion effects. The change in the solute uptake is dependent on *t*^1/2^ as indicated by the Weber-Morris model (Fig. 15). A single method is not always diligent in charge of the kinetics of adsorption. We find that there are many levels of linearity across all solute concentrations. The adsorption frequency is high at lower initial concentrations (75 ppm) and lower temperatures. The rate will subsequently follow a linear decreasing curve until it stabilises after a gradual decrease.

**Fig. 16:**
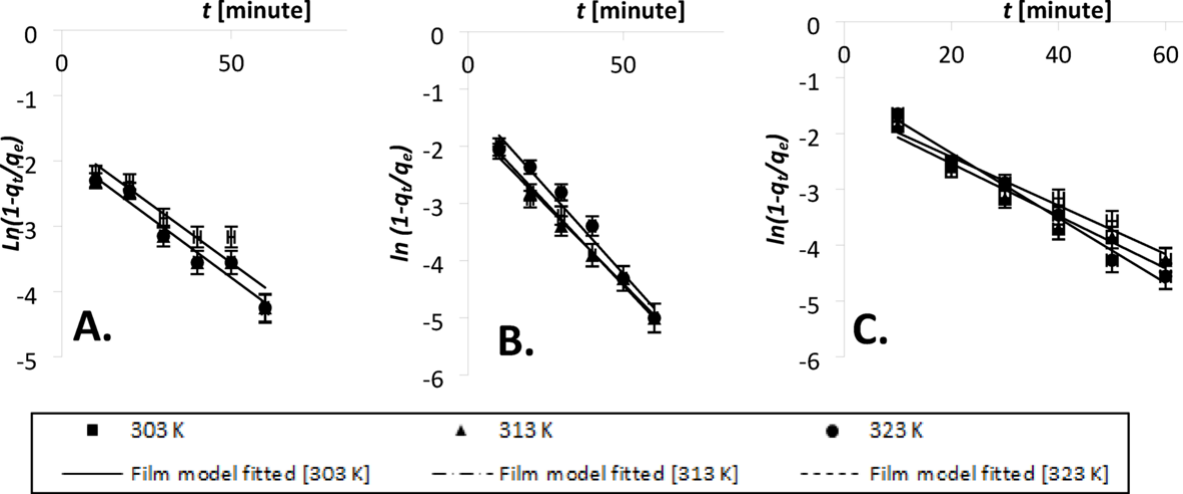
Kinetics data fitted to the Film diffusion model with preliminary concentration of AB-113 dye: (**A**) 75 ppm, (**B**) 150 ppm, (**C**) 300 ppm.

At high temperature, though, the rate is more linear. The model of film diffusion as it approaches higher temperatures shows that the adsorption rate changes less prominently with solute concentration (300 ppm). This is due to high fidelity to the model as demonstrated by high *R*^2^ and large values of *χ*^2^, resulting in a liquid film diffusion constant *R*^*'*^ be (Table 6 and Fig. 16) as indicated by the data in Fig. 15. Diffusional inherent limits result in minor impediments and an increased rate of obstructive adsorption. We can conclude a rate limit that is created by diffusion. A film of the solute is then quickly adsorbed onto the micro-particle surface, which changes the rates of solute uptake and prevents subsequent diffusion.

### Adsorption thermodynamics

**Fig. 17:**
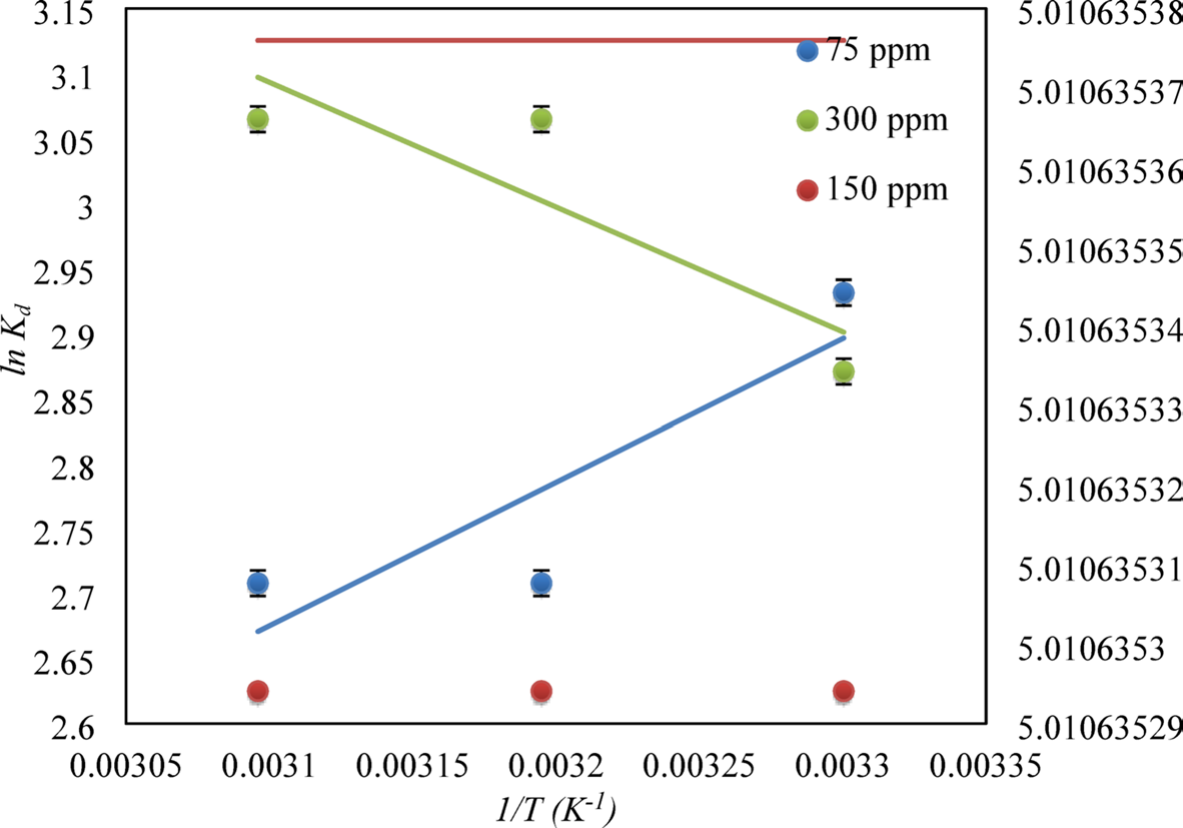
Thermodynamic constant at equilibrium v/s 1/T.

**Fig. 18:**
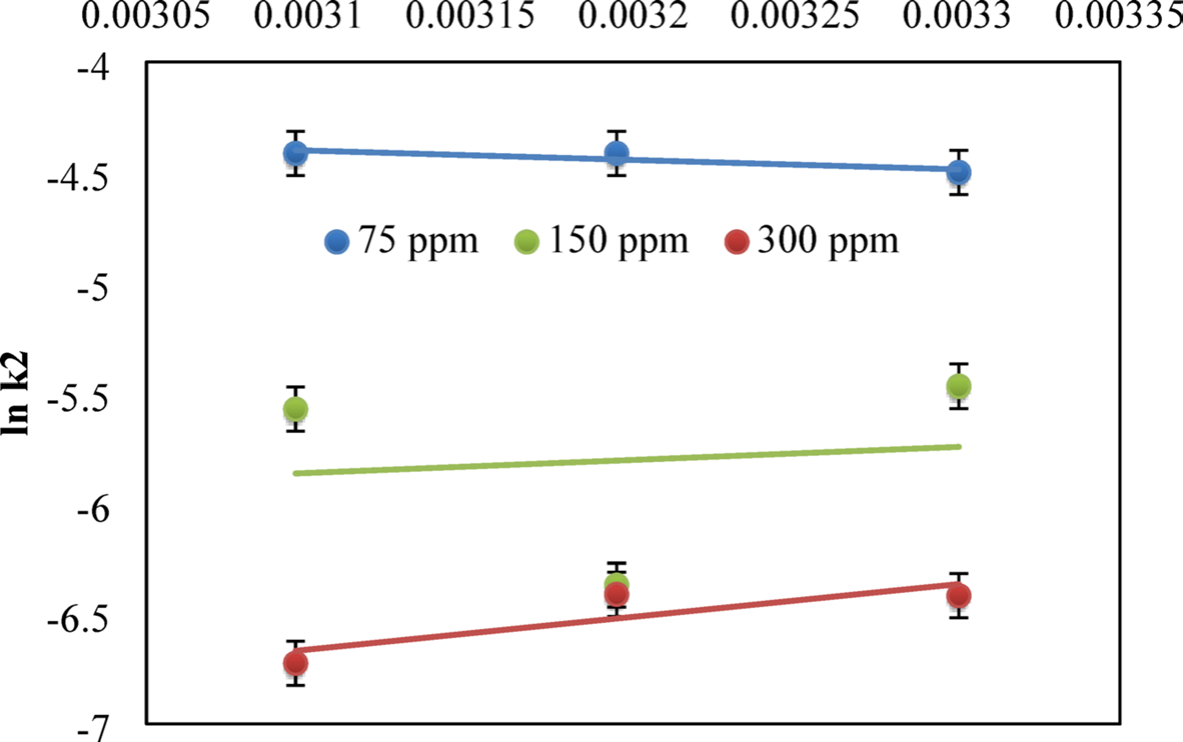
Kinetic constant of pseudo-second-order v/s 1/T.

The primary variables to be considered when designing the interface method are energy and entropy. Gibbs free energy change is commonly used as a parameter to indicate the spontaneity of the adsorption process; the standard Gibbs free energy change, ΔG°, should be considered. When the free energy change (Δ*G°*) of adsorption is negative, significant adsorption will occur. *∆H°*, *∆S°*, and *E*_*a*_ can be computed using the Van’t Hoff plots of ln (*K*_*d*_) and ln (*K*_2_) versus 1/T, as shown in Figs. 17 and 18, respectively.

Estimates of the thermodynamic parameters are provided in Table 7. In the situation where the value of *∆H*^*0*^ is positive and that of *∆G*^0^ is negative, the adsorption course appears realistic and impulsive, thereby implying that the adsorption is endothermic. It is an advantageous and spontaneous trend in the AB 113 adsorption on MNC, as all calculated ∆G^0^ values are negative at all studied temperatures. The values of the *∆G*^0^ degree show a negative relationship with temperature, indicating that adsorption increases with temperature. This is due to the strong binding of AB113 to the adsorbent. The lower values of *∆H*^0^ indicate that the adsorption is physical, as the standard enthalpy change for a chemical reaction is above 200 kJ mol^−1^. The Arrhenius equation and the kinetic constant from the pseudo-second-order model were used to estimate the activation energy of the adsorption process at room temperature and at elevated temperatures. 75, 150, and 300 ppm of starting concentrations have further corroborated this, ranging from ~ -12.32 to 3.54 kJ mol^−1^.

**Table 7:** AB113-MNC thermodynamic assemblies.

Initial concentration (ppm)	Temperature (*K*)	Δ*G°* (kJ mol^−1^)	Δ*S°* (J mol^−1^ K^−1^)	Δ*H°* (kJ mol^−1^)	ln *A*	*E*_*a*_ (kJ mol^−1^)
75	303	-7.38	-6.19	-9.17	-3.08	3.54
	313	-7.05				
	323	-7.27				
150	303	-12.62	0	-0.04	-7.69	-4.90
	313	-13.04				
	323	-13.45				
300	303	-7.23	50.46	7.98	-11.25	-12.32
	313	-7.97				
	323	-8.23				

### Statistical optimisation by fractional factorial experimental design

**Fig. 19:**
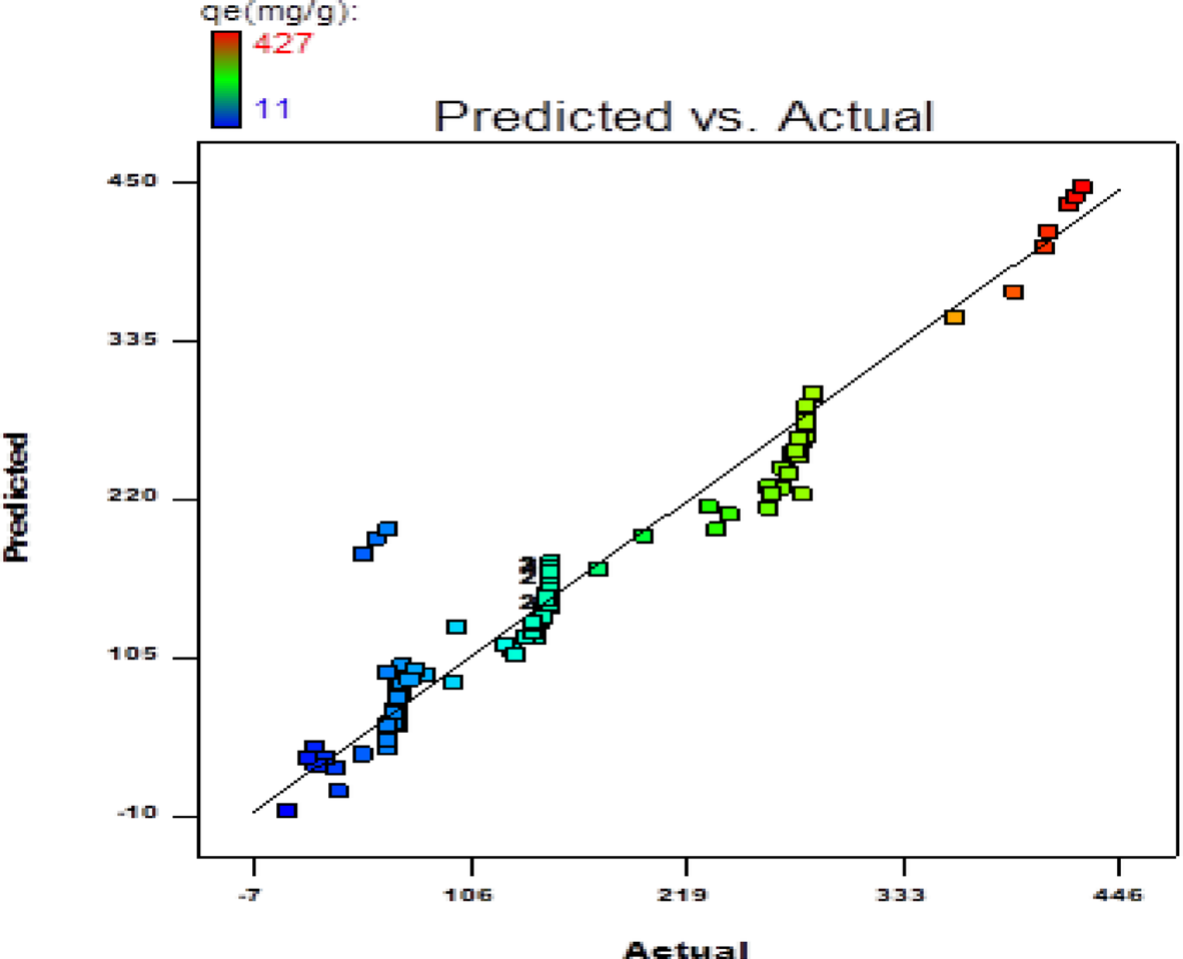
Statistical plot actual versus predictive results.

The effects of each of the five independent variables were considered separately and jointly by using several combinations of the five variables in experiments. The significance of each factor’s individual and combined impacts is clearly shown by the quadratic regression analysis of variance (Table 8). Factor significance was defined by a 95 per cent confidence interval p-value that is less than or equal to 0.05 per cent. All other variables in the study are not significant, but A, C, D, AC, A^2^, C^2^, and D^2^ of the model are significant. Each of AD, AE, BD, BF, BE, CD, CE, CF, and DE have no cross-products. The RSM model is very important, as indicated by its model F-Value of 84.0. The anticipated *R*^2^ value of 82.8% and the updated *R*^*2*^ value of 90.8% are reasonably good. The model* R*^2^ value of 90.8% and the high coefficient of variance (19.4%) indicate that it can be used to investigate the design space. The correlation between experimental and expected responses is good because the comparison graph of the actual values against the expected values (Fig. 19) indicates that the approximations are close to the expected values. The regression obtained during the study is presented below.

$$\begin{gathered} {\mathrm{Adsorption}} = {246}.{8} + {87}.{3}*{\mathrm{A}} + {18}.{9}*{\mathrm{B}} + {129}.{2}*{\mathrm{C}} \hfill \\ \quad \quad \quad \quad \quad \quad \; - {92}.{5}*{\mathrm{D}} - {4}.{5}*{\mathrm{AB}} + {88}.{6}*{\mathrm{AC}} + {28}.{9}*{\mathrm{BC}} \hfill \\ \quad \quad \quad \quad \quad \quad \; - {55}.{3}*{\mathrm{A}}^{{2}} {3}.{6}*{\mathrm{B}}^{{2}} - {225}.{8}*{\mathrm{C}}^{{2}} + {1}0{6}.{7}*{\mathrm{D}}^{{2}} - {27}.{4}*{\mathrm{E}}^{{2}} \hfill \\ \end{gathered}$$

**Table 8:** ANOVA table.

Source	Sum of squares	Degree of freedom	Mean square	F value	P-value
Model	1,032,404.8	13	79,415.8	84.0	< 0.001**
A	15,362.0	1	15,362.0	16.2	< 0.001**
B	865.9	1	865.9	0.9	0.3410
C	25,121.5	1	25,121.5	26.6	< 0.001**
D	35,647.4	1	35,647.4	37.7	< 0.001**
E	0.0	1	0.0	0.0	< 0.001**
AB	103.3	1	103.3	0.1	0.7417
AC	10,517.6	1	10,517.6	11.1	< 0.001**
BC	1215.6	1	1215.6	1.3	0.2596
A^2^	21,411.0	1	21,411.0	22.6	< 0.001**
B^2^	165.0	1	165.0	0.2	0.6770
C^2^	91,414.9	1	91,414.9	96.7	< 0.001**
D^2^	12,722.9	1	12,722.9	13.5	< 0.001**
E^2^	1401.6	1	1401.6	1.5	0.2264
Residual	90,761.6	96	945.4		
Total	1,123,166.4	109

Significant figures: + Suggestive significance (*p*-value: 0.05 < *p* < 0.10). *Moderately significant (*p*-value: 0.01 < *p* ≤ 0.05). **Strongly significant (*p-*value: *p* ≤ 0.01).

**Fig. 20:**
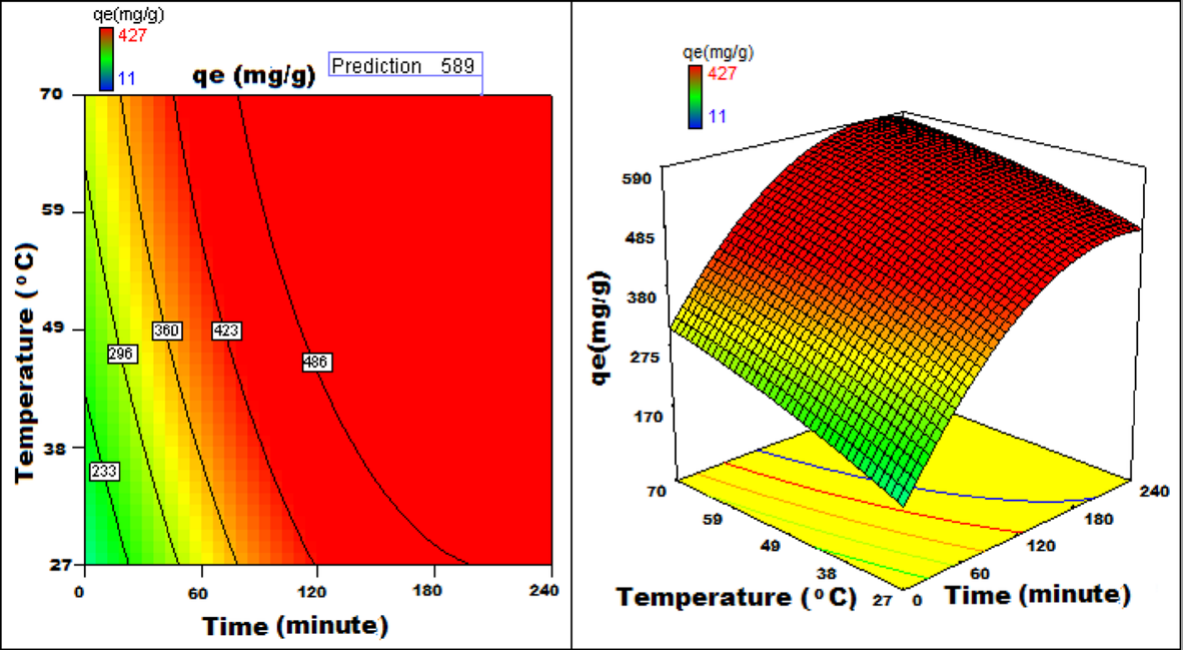
Variability of adsorption capacity with temperature versus time.

Maximising the second-order polynomial equation, whose interaction factors were calculated through multiple regression analysis, yielded the optimum values of the variables as a function of FFED. In the best-case scenario, supplemented with 0.500 g L^−1^ powder, the initial dye concentration was 858 mg L^−1^, the optimal adsorption time was 204 min, orbital shaking was at 165 rpm, and the temperature was 70 °C. The maximum adsorption capacity, determined by statistical optimisation, was 589 mg g^−1^. The second operation of the statistical optimisation process consisted of analysing 3D response surface plots and 2D contour plots, taking into consideration variations in the two independent variables. The interaction effects between the two factors and the remaining parameters were identified during the process. Statistical process optimisation may identify which of the two conditions is superior and how the process parameters affect adsorption, depending on the parameter ranges. It is observed that time positively correlates with adsorption capacity, as shown by three-dimensional plots over time and across all other variables. Adsorption can be accelerated by increasing the temperature, time, and dye concentration. The required time is 204 min to maximise adsorption. Temperature changes positively affect the adsorption capacity. The higher the temperature, the greater the adsorption capacity. As demonstrated, the adsorption capacity increases with increasing original dye concentration. It was found that the dye concentration increases with increasing adsorption. Considering the plot of temperature against other independent variables, it can be seen that temperature positively affects the reaction with all the components. A surge in the initial concentration will be advantageous for adsorption, as shown by graphs illustrating the relationship between the initial dye concentration and other factors. It is at a starting concentration of 858 of dye that adsorption reaches a maximum. Figure 20 illustrates the interactive influence of contact time and temperature on the adsorption capacity (*q*_*e*_, mg g^−1^) of AB113 on montmorillonite nanoclay, depicted as a 2D contour map (left) and a 3D response surface (right) derived via quadratic regression/RSM optimisation: *q*_*e*_ escalates significantly over time from the initial phase to approximately the mid-late phase (indicating a swift occupation of accessible sites succeeded by a gradual approach to saturation), and it also rises with temperature within the examined range, resulting in an ascending “surface” and contour bands that shift towards elevated *q*_*e*_ at extended durations and increased temperatures; The plot illustrates that the optimal performance region occurs at an extended contact time (approximately 200 min) with a moderate temperature (around 30–50 °C in the experiment; the model peak zone is indicated at the upper limit of the examined range) where *q*_*e*_ approaches the model-predicted maximum (approximately 589 mg g^−1^). Conversely, shorter contact times—even at elevated temperatures—fail to achieve this capacity due to insufficient progress towards equilibrium and ongoing intra-particle/film mass-transfer limitations that hinder uptake.

**Fig. 21:**
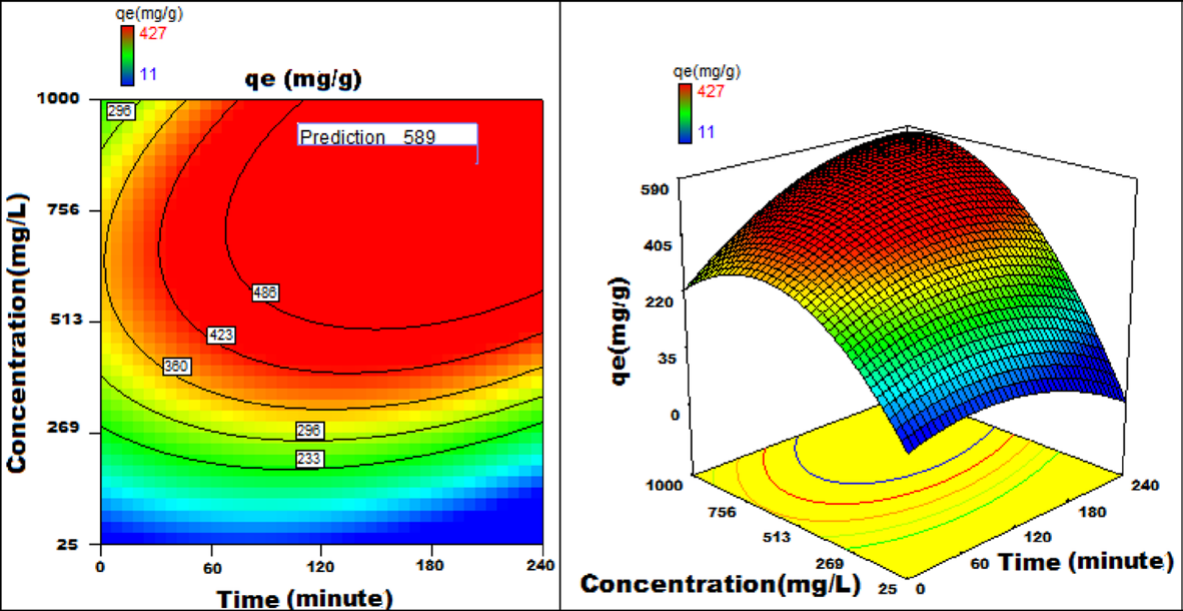
Variability of adsorption capacity with time versus concentration.

Figure 21 depicts, via the paired contour plot (2D) and response surface (3D), the simultaneous variation of adsorption capacity (*q*_*e*_, mg g^−1^) with respect to contact time and initial AB113 concentration. It distinctly demonstrates that *q*_*e*_ significantly increases as concentration escalates from low to mid-high levels and as time progresses into the extended-contact region, resulting in a broad high-*q*_*e*_ “plateau,” where the model forecasts a maximum of approximately 589 mg g^−1^ at around 200 min and 850–900 mg L^−1^.

**Fig. 22:**
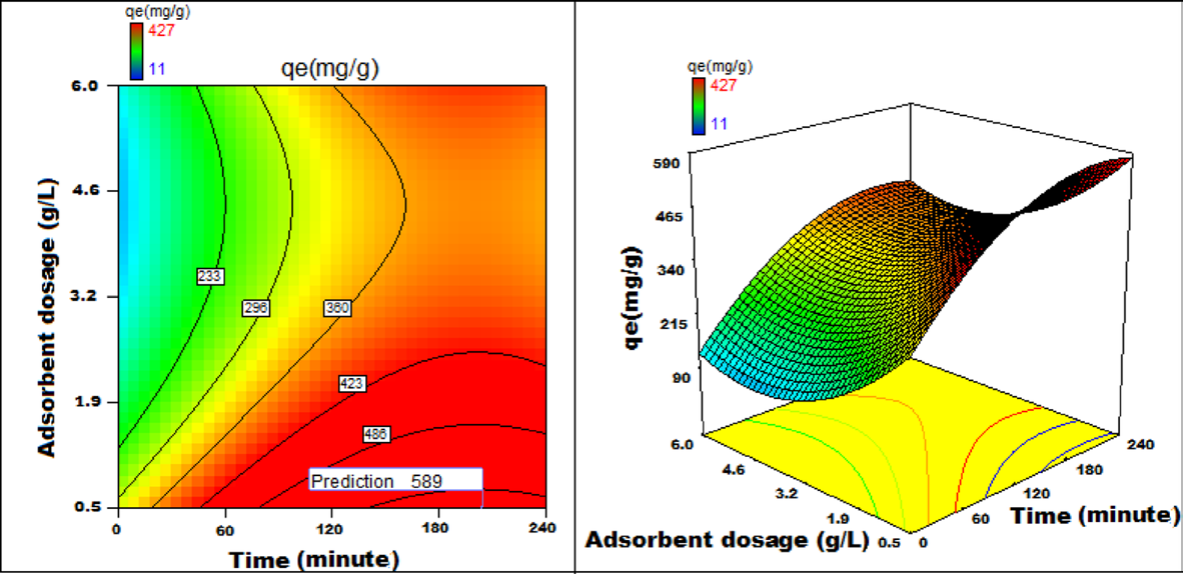
Variability of adsorption capacity with time versus adsorbent dosage.

Following Fig. 22 depicts the synergistic influence of contact duration and adsorbent quantity on the adsorption capacity (*q*_*e*_, mg g^−1^) of AB113 onto montmorillonite nanoclay, utilising 2D contour and 3D response surface plots generated from the RSM model. The adsorption capacity rises with contact time as active sites become increasingly occupied and dye molecule diffusion within the clay structure improves. Conversely, *q*_*e*_ diminishes with increasing adsorbent dose, with peak capabilities noted at a low MNC loading of around 0.5 g L^−1^, coupled with an extended contact duration of around 200 min, when the anticipated maximum of approximately 589 mg g^−1^ is reached. The inverse dose effect occurs when increased adsorbent loadings provide more surface area than available dye molecules, resulting in site underutilization, particle aggregation, and overlap of adsorption sites. The dye absorption per unit mass of adsorbent diminishes, despite a potential enhancement in total removal efficiency.

**Fig. 23:**
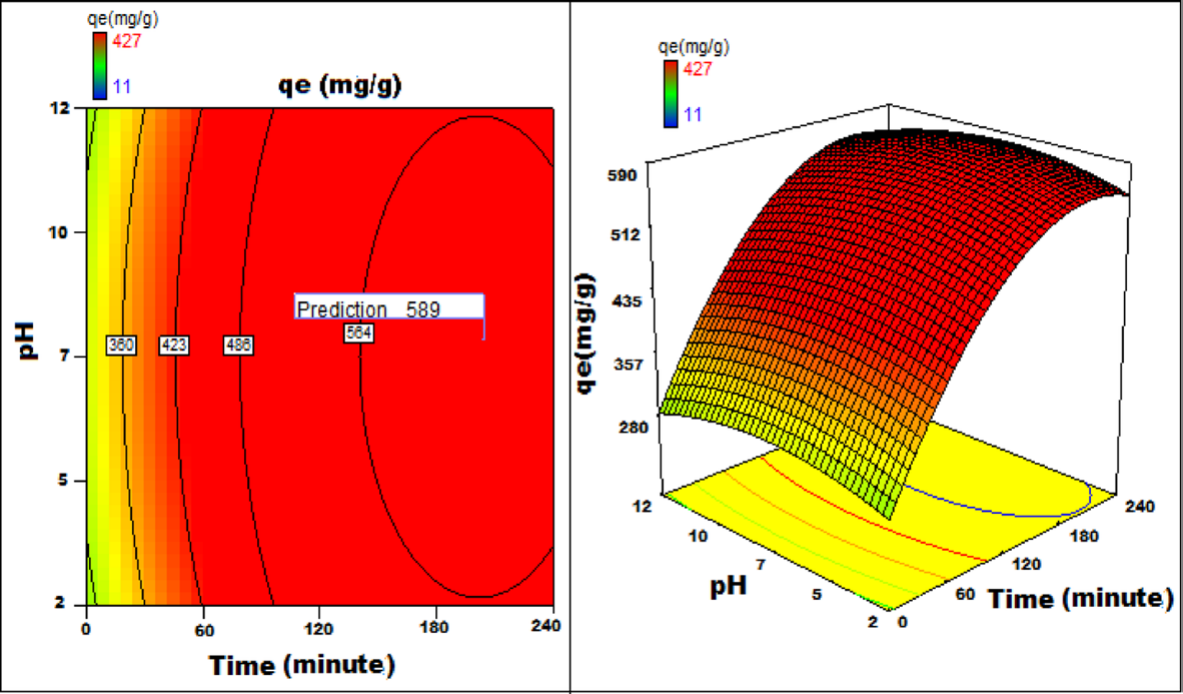
Variability of adsorption capacity with time versus pH.

Figure 23 illustrates the synergistic influence of contact duration and solution pH on the adsorption capacity (*q*_*e*_, mg g^−1^) of AB113 onto montmorillonite nanoclay, utilising contour and response surface plots. The adsorption capacity significantly increases over time, reaching a maximum at prolonged contact times of around 180–220 min.

In contrast, *q*_*e*_ remains comparatively constant over a broad pH range from 2 to 12, with only a slight optimum at neutral pH. This pattern suggests that the adsorption process is predominantly pH-independent, aligning with the point-of-zero-charge of MNC and the preeminent influence of physical interactions.

**Fig. 24:**
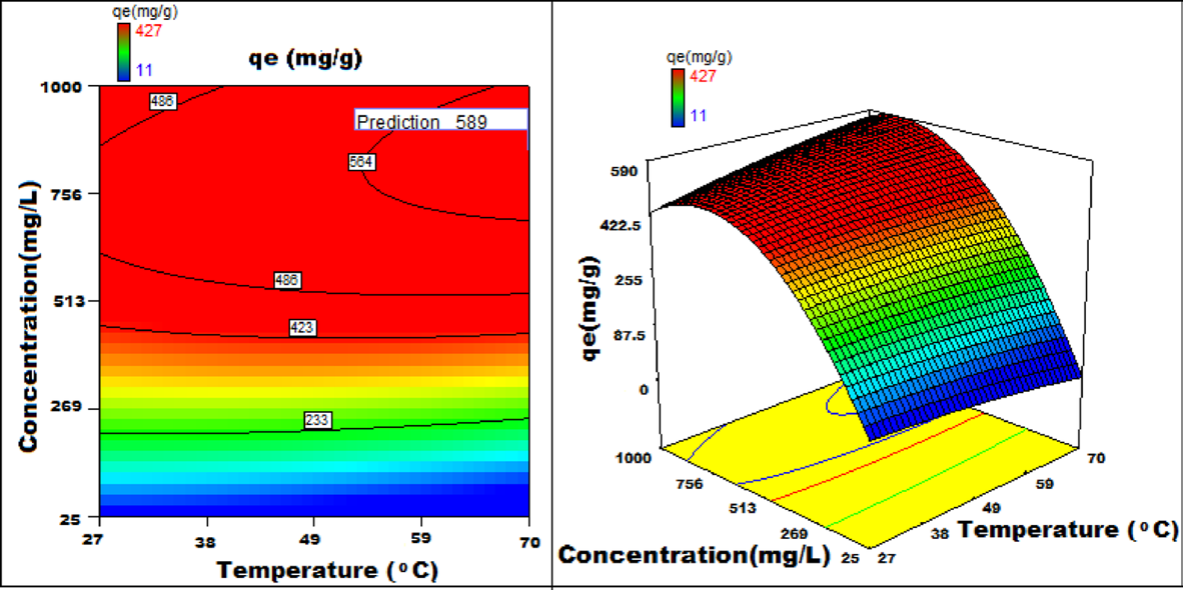
Variability of adsorption capacity with temperature versus concentration.

Figure 23 illustrates the minimal impact of pH on adsorption efficacy, underscoring the process’s resilience across a broad pH range, whereas Fig. 24 examines the combined effects of temperature and initial dye concentration on adsorption capacity. Figure 24 illustrates that *q*_*e*_ significantly increases with higher starting AB113 concentrations and is further enhanced by mild temperature changes, with maximum adsorption capacity recorded at elevated dye concentrations, where the mass transfer driving force is most substantial. At low concentrations, adsorption capacity is constrained, irrespective of temperature, due to insufficient dye availability. The response surface suggests that temperature has a secondary yet supporting function by increasing molecule mobility and diffusion, therefore promoting more efficient occupancy of active sites at elevated concentrations.

**Fig. 25:**
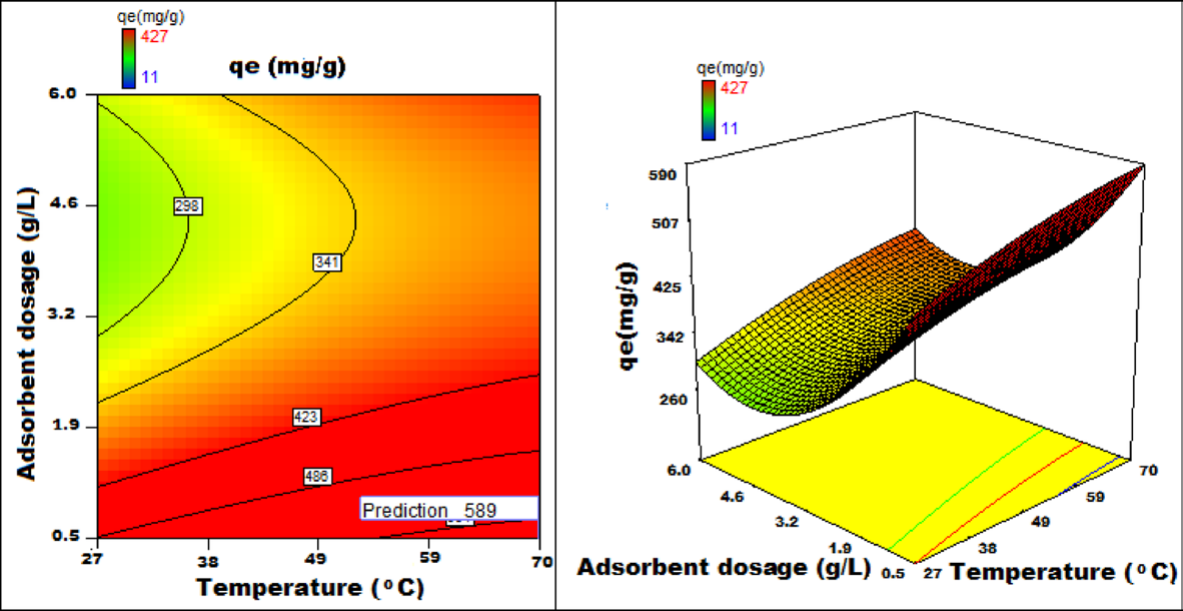
Variability of adsorption capacity with temperature versus absorbent dosage.

**Fig. 26:**
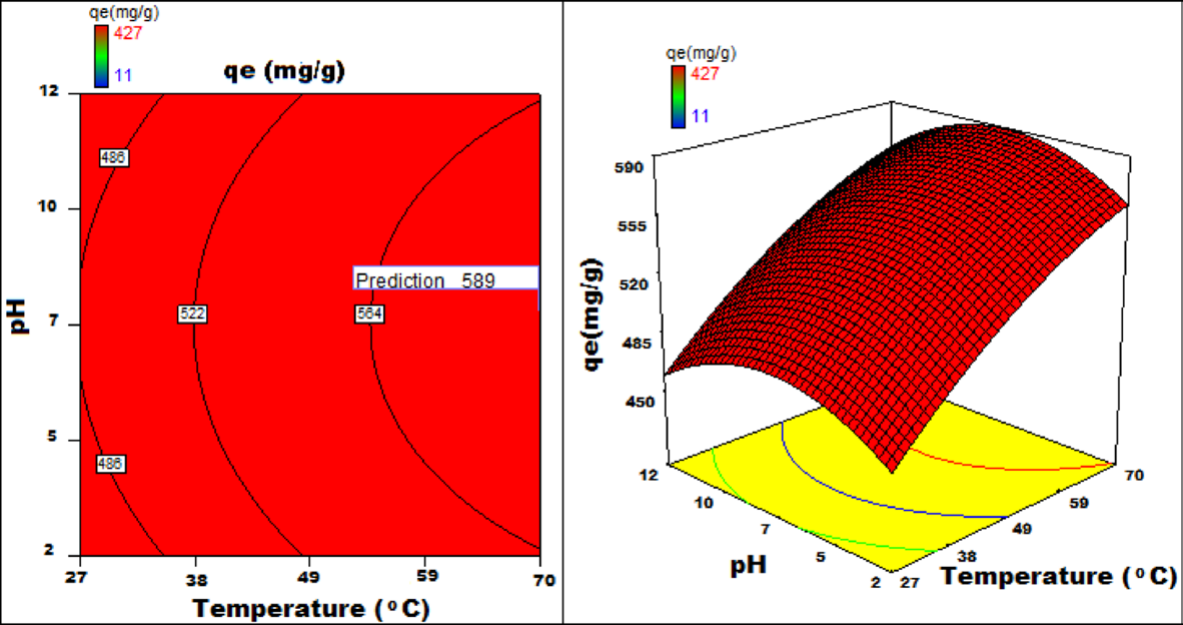
Variability of adsorption capacity with temperature versus pH.

Figure 25 depicts the interaction influence of temperature and adsorbent dose on the adsorption capacity (*q*_*e*_, mg g^−1^) of AB113 onto montmorillonite nanoclay. The adsorption capacity increases with temperature, indicating greater dye mobility and enhanced mass transfer at higher temperatures. Conversely, *q*_*e*_ diminishes as the adsorbent dose increases, with greater capabilities shown at lower MNC loadings. This pattern indicates the underutilization of adsorption sites and potential particle aggregation at elevated doses, which diminishes dye absorption per unit mass of adsorbent despite an increased total surface area. The response surface indicates that temperature somewhat mitigates this effect by enhancing site occupancy, especially at low doses. Figure 26 shows the combined effect of temperature and pH on the adsorption capacity (*q*_*e*_, mg g^−1^) of AB113 onto montmorillonite nanoclay. Adsorption capacity increases with temperature, while pH has only a minor influence over the broad range of 2–12. High *q*_*e*_ values are maintained across acidic, neutral, and alkaline conditions, with a shallow maximum near neutral pH.

**Fig. 27:**
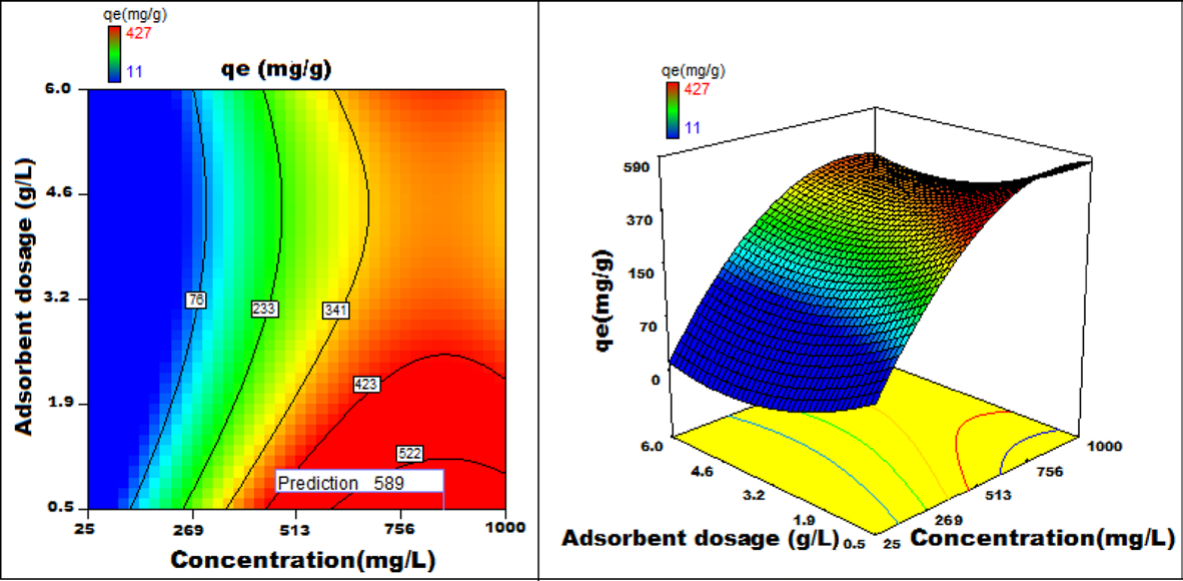
Variability of adsorption capacity with adsorbent dosage versus concentration.

This figure highlights the pH-independent nature of the adsorption process and confirms that temperature plays a more influential role than pH. It demonstrates the system’s operational robustness and supports its suitability for real textile effluents without requiring strict pH control. The subsequent image, Fig. 27, illustrates the interaction influence of adsorbent dose and starting dye concentration on the adsorption capacity (*q*_*e*_, mg g^−1^) of AB113 onto montmorillonite nanoclay. The adsorption capacity significantly increases with rising dye concentration, indicating an increased driving force for mass transfer. Conversely, *q*_*e*_ diminishes as the adsorbent dose increases, with optimal capacity achieved at low MNC loading and elevated dye concentration. This behaviour indicates underutilization of adsorption sites and particle agglomeration at elevated doses.

**Fig. 28:**
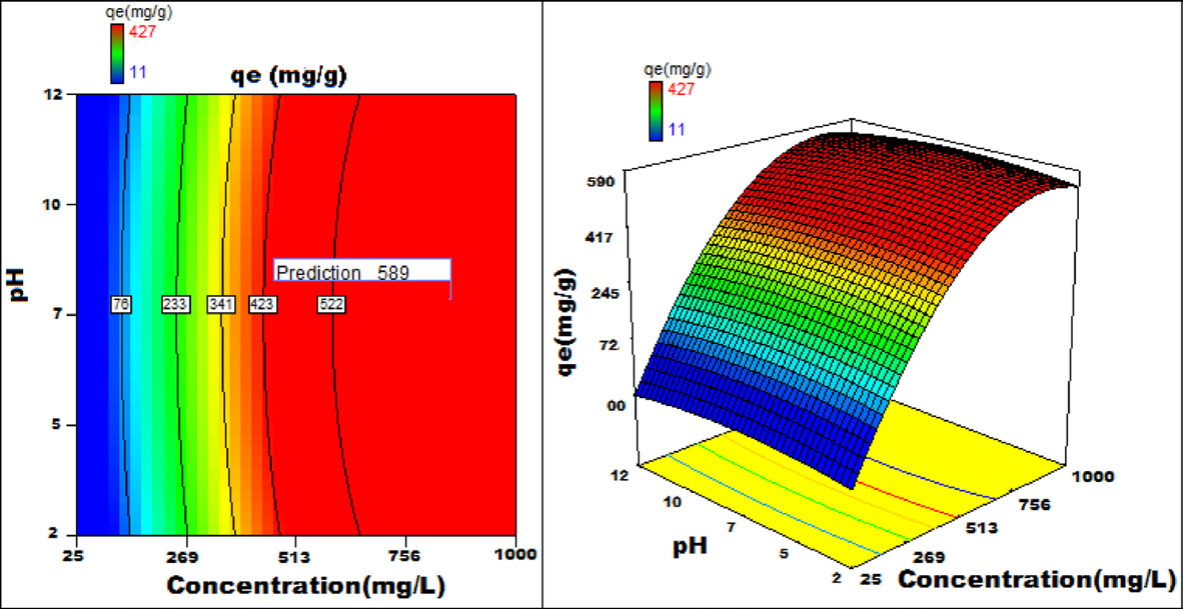
Variability of adsorption capacity with pH versus concentration.

Figure 28 illustrates the synergistic impact of solution pH and starting dye concentration on the adsorption capacity (*q*_*e*_, mg g^−1^) of AB113 onto montmorillonite nanoclay. The adsorption capacity significantly escalates with rising dye concentration, although pH has little effect over the extensive range of 2–12. Elevated *q*_*e*_ values are sustained throughout acidic, neutral, and alkaline environments, with optimal adsorption observed at high concentrations regardless of pH. This graphic illustrates that dye concentration is the primary variable, whereas pH plays a subordinate role. This validates the pH-resistance of the adsorption technique and endorses its suitability for real textile effluents without pH modification. Ultimately, Figs. 20, 21, 22, 23, 24, 25, 26, 27 and 28 show the effects of the two factors on biosorption using surface and contour plots. The quadratic model generated in course optimisation has proved sufficient to forecast the maximum adsorption capacity, while also revealing the relationships among independent parameters and how they exert their impacts on the adsorption process. The process optimisation increases adsorption to 589 mg g^−1^, compared to 149 mg g^−1^. It exceeds 395 per cent, and that is a good commercialisation proposal.

### Experimentation using the proposed method on textile industrial effluent [TIE]

Many operations in the textile industry generate wastewater with a broad range of compositions, viz., high suspended solids content, high adsorption pH, rapid pH changes, sudden temperature changes, intense colour, and excessive COD levels^62^. It is challenging to measure a specific colour in industrial effluent due to the matrix effect^63^. The responsible officials do not disclose the kinds of dyes used in the sector for security reasons, nor their procedures. The blue TIE that was gathered isn’t always AB113 dye. A simple method was developed to compare the removal of AB113 dye in both textile-sector water and wastewater.

#### Results of the experiments with textile industrial effluent

Initial trial experiments indicate that the most desirable outcomes occur at a doubling: a fivefold increase in the adsorbent, a tenfold increase in the adsorbate, and an order-of-magnitude increase in the solution volume. The absorbance of Solution 2 was more than 4% lower than that of Solution 1, which was an interesting finding. This finding might be due to the dye being absorbed by multiple unidentified TIE components. In addition, it was noted that the capacity to remove the dye in TIE was enhanced by introducing new adsorbent samples every 15 min. The dye and associated chemicals were recovered at 96%, 98%, and 99% from Solution 2 after 15, 30, and 45 min, respectively. Kinetic measurements, which show the solute rapidly sorbs onto the particle surface as a film that then retards fluid flow, are consistent with this discovery and result in a shift in absorption rates.

Using 1, 2, and 5 L of Solution 2, the experiment was performed with increasing volumes by multiplying the amount of MNC by 10 g, 20 g, and 50 g in HDPE beakers. As mentioned earlier, the liquids were thoroughly mixed using a magnetic stirrer, and the process was repeated. The findings were nearly the same. Each experiment was repeated thrice, and the average of the three values was calculated. The results were shared. The coefficients of variation for all outcomes were within 2%.

The primary restriction within this study is that, more often than not, industrial effluents exhibit a wide range of variables that cannot be addressed straightforwardly from the initial data; much more exhaustive pilot-scale research is required to elicit the outcome. The realisation that the negative aspects of the scale were identified and that the enhanced scale experimental findings would still prove the potentiality and validity of the procedure is sufficient to believe that the principles of the approach would come into use when applied to industries on a larger scale. To conclude, scaled-up versions of the experiment by a factor of 3 or more have shown promising results compared to the original setup.

### Regeneration of the adsorbent and cost analysis

The dye-loaded MNC could be regenerated based on the adsorbed material, enabling reuse. The dye-loaded MNC should not be regenerated because the solvent costs incurred during the process would be quite high compared to the costs of the adsorbents utilised. In addition, it will boost the E-factor^64^, which is undesirable, as the world cannot tolerate any additional environmental pollutants. To eliminate waste materials in the process, our lab is currently developing another method for making thermosets.

### Proposed synergistic adsorption mechanism of AB113 on MNC

**Fig. 29:**
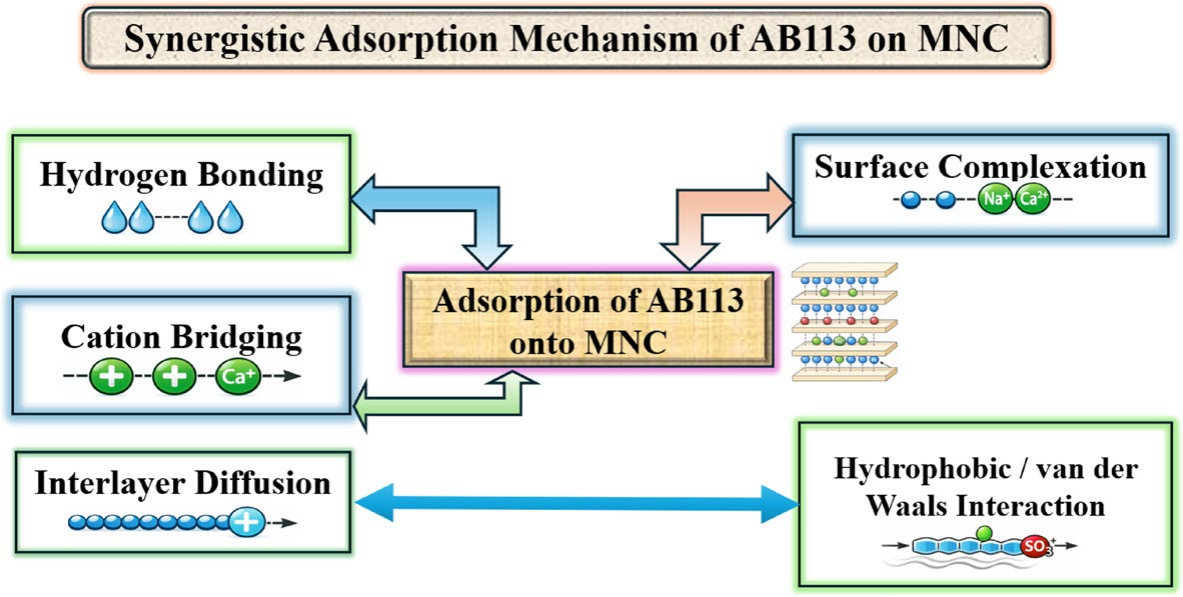
Synergistic adsorption mechanism of AB113 onto MNC.

A schematic representation of the possible adsorption mechanism of AB113 onto MNC is proposed to explain the observed adsorption behavior (Fig. 29). The adsorption appears to be governed by a synergistic combination of interactions rather than by a single mechanism. MNC provides layered silicate surfaces, exchangeable interlayer cations, and edge hydroxyl groups, whereas AB113 contains a large aromatic bis-azo framework with sulfonate and nitrogen-containing functionalities. Accordingly, AB113 adsorption may proceed through: (i) hydrogen bonding between MNC hydroxyl groups and dye functional groups; (ii) cation bridging between sulfonate groups of AB113 and exchangeable interlayer cations of MNC; (iii) weak surface complexation at edge sites; (iv) van der Waals/hydrophobic interactions between the aromatic dye structure and the siloxane surface; and (v) possible interlayer diffusion or anchoring within accessible clay galleries. The cooperative action of these mechanisms plausibly accounts for the high adsorption efficiency and the negligible pH dependence observed over the pH range 2–12.

#### Limitations and practical implementation challenges

While the present study demonstrates the effectiveness of MNC as a sustainable adsorbent for the removal of Acid Blue 113 (AB113), several limitations and practical challenges must be acknowledged. First, the experiments were conducted under controlled laboratory conditions, which may not fully replicate the complexity of real textile industrial effluents. In practice, wastewater streams contain a diverse mixture of dyes, salts, surfactants, heavy metals, and auxiliary chemicals, which may influence adsorption performance through competitive interactions and matrix effects. Second, although the adsorption process exhibited remarkable robustness across a wide pH (2–12) and temperature (30–50 °C) range, the influence of high ionic strength and multicomponent systems was not systematically evaluated and warrants further investigation. Third, the study does not extensively explore adsorbent regeneration and reuse. While regeneration was considered less economically favourable due to solvent costs and environmental concerns, the long-term operational feasibility of single-use adsorbents at scale requires further assessment. Fourth, scale-up challenges, including mass transfer limitations, hydrodynamic conditions, and reactor design, may influence adsorption efficiency in industrial applications. Although preliminary scale-up experiments showed promising trends, detailed pilot-scale validation is necessary to confirm process viability. Finally, the handling and utilisation of dye-loaded sludge, although addressed through proposed valorisation into composite materials, require further optimisation and lifecycle assessment to ensure environmental safety and economic feasibility. Despite these limitations, the present study provides a strong foundation for the development of robust, low-cost, and sustainable adsorption systems, and highlights key directions for future research toward real-world implementation.

## Conclusion

Many nations’ textile industries have adopted unsuitable and unsustainable processes, leading to technological divide and severe environmental problems. We must investigate and identify more efficient methods for using industrial effluent from recovered textiles and for reducing the textile sector’s water footprint. With its complex composition and high concentration of hazardous chemicals, Textile Industrial Effluent (TIE) is a challenging waste product. The search for ecologically friendly ways to remove toxicants from TIE has led to investigating nanoparticles as adsorbents in various treatment systems—the textile industry benefits from this study’s promotion of sustainability. The two primary factors are recycling the TIE for future use and the impact of water use. Customised lab-based studies illustrate the circular economy paradigm by employing Montmorillonite Nanoclay to remediate textile dyes in a way that complies with sustainability and value-adding principles. It was reported that MNC is an excellent adsorbent for AB113 in aqueous solution. As part of the process optimisation, the adsorption capacity of MNC was measured using a quadratic model derived from the Vieth Sladek isotherm. It was determined to exude 589 mg g^−1^. Adsorption of B113 was nearly spontaneous and endothermic. These outcomes were related to the pseudo-second-order kinetic model. The optimal fit was obtained during the kinetic investigations, as a result of which the pseudo-second-order model is suitable. Physical processes involved mass transfer mediated by intraparticle diffusion. AB113 was adsorbed onto MNC to battle pollution, offering a more effective, affordable, and long-lasting solution, as confirmed by the SEM and FTIR spectra. The results of the experiments demonstrate that TIE’s greywater pollution can be reduced or converted into blue water.

The strict policies of regulatory bodies enhanced efficiency in the textile industry, and the high cost of activated charcoal has seen MNC back on the scene, which is widely accessible, less costly, and easier to use than charcoal-based adsorbents. MNC maximizes water security, minimises greywater footprints, and lowers the E-factor as an efficient adsorbent. Carbon footprints are reduced when dye-adsorbed MNC (sludge) is used as a raw material to create composite products from plastic trash. Thus, it is conceivable to converge the demands of a circular economy by addressing the unresolved issue of sludge disposal. To summarise, the new study introduces a further paradigm for use, combining sustainability and the water footprint paradigm. Commercially, any implementation of the approach will reduce carbon and water footprints and is quite financially advantageous. Additionally, our research will present an alternative strategy for addressing resource depletion and environmental pollution in the twenty-first century. The project’s creators hope that by reducing carbon and water footprints and creating new opportunities aligned with the circular economy’s awareness, their work will open new avenues for green technology and sustainability.

## Data Availability

All data generated or analysed during this study are included in this published article.
